# Bacterial co-infection and antibiotic stewardship in patients with COVID-19: a systematic review and meta-analysis

**DOI:** 10.1186/s12879-022-07942-x

**Published:** 2023-01-09

**Authors:** Maria Calderon, Grace Gysin, Akash Gujjar, Ashleigh McMaster, Lisa King, Daniel Comandé, Ewan Hunter, Brendan Payne

**Affiliations:** 1 Department of Infection and Tropical Medicine, Newcastle-Upon-Tyne Hospitals NHS Foundation Trust, Royal Victoria Infirmary, Queen Victoria Rd., Newcastle-Upon-Tyne, NE1 4LP UK; 2 Translational and Clinical Research Institute, Newcastle University, Newcastle-Upon-Tyne, NE1 7RU UK; 3 Instituto de Efectividad Clinica y Sanitaria, Emilio Ravignani 2024 (C1414CPV), Buenos Aires, Argentina; 4 School of Medicine, Newcastle University, Newcastle-Upon-Tyne, UK

**Keywords:** Bacterial co-infection, COVID-19, Antibiotic stewardship

## Abstract

**Introduction:**

Understanding the proportion of patients with COVID-19 who have respiratory bacterial co-infections and the responsible pathogens is important for managing COVID-19 effectively while ensuring responsible antibiotic use.

**Objective:**

To estimate the frequency of bacterial co-infection in COVID-19 hospitalized patients and of antibiotic prescribing during the early pandemic period and to appraise the use of antibiotic stewardship criteria.

**Methods:**

Systematic review and meta-analysis was performed using major databases up to May 5, 2021. We included studies that reported proportion/prevalence of bacterial co-infection in hospitalized COVID-19 patients and use of antibiotics. Where available, data on duration and type of antibiotics, adverse events, and any information about antibiotic stewardship policies were also collected.

**Results:**

We retrieved 6,798 studies and included 85 studies with data from more than 30,000 patients. The overall prevalence of bacterial co-infection was 11% (95% CI 8% to 16%; 70 studies). When only confirmed bacterial co-infections were included the prevalence was 4% (95% CI 3% to 6%; 20 studies). Overall antibiotic use was 60% (95% CI 52% to 68%; 52 studies). Empirical antibiotic use rate was 62% (95% CI 55% to 69%; 11 studies). Few studies described criteria for stopping antibiotics.

**Conclusion:**

There is currently insufficient evidence to support widespread empirical use of antibiotics in most hospitalised patients with COVID-19, as the overall proportion of bacterial co-infection is low. Furthermore, as the use of antibiotics during the study period appears to have been largely empirical, clinical guidelines to promote and support more targeted administration of antibiotics in patients admitted to hospital with COVID-19 are required.

**Supplementary Information:**

The online version contains supplementary material available at 10.1186/s12879-022-07942-x.

## Introduction

The COVID-19 pandemic has impacted health systems worldwide, with SARS-CoV-2 infection being implicated in more than 6 million deaths to date [1, 2]. Some clinical guidelines have recommended empirical antibiotic therapy to treat suspected bacterial respiratory co-infection in COVID-19 patients, and tools to support and promote antibiotic stewardship in this population are therefore needed [3, 4].

Distinguishing between viral pneumonia and bacterial co-infection at presentation and during the course of COVID-19 disease can be challenging due to various similarities, including characteristically high inflammatory markers and the frequent presence of pulmonary infiltrates on chest X-ray or computed tomography (CT) imaging [5]. There is therefore potential for considerable overuse of antibiotics in the management of COVID-19 pneumonia, with the attendant risk of an increase in the prevalence of antimicrobial resistance in affected populations. Given the current pandemic context, the implications of this for public health and health systems are likely to be considerable. Clinical guidelines to support the most effective treatment for patients while promoting the responsible use of antibiotics should be informed by an understanding of what proportion of patients admitted to hospital with COVID-19 pneumonia have confirmed acute respiratory bacterial co-infection and of the commonly associated pathogens.

We performed a systematic review to estimate the frequency of confirmed bacterial co-infection in patients admitted to hospital with COVID-19 pneumonitis, the frequency of empirical antibiotic use in this patient group, and to identify any antibiotic stewardship criteria that have been used during the COVID-19 pandemic to date.

## Methods

We registered the review protocol at the PROSPERO international prospective register of systematic reviews (CRD 42020181215). We followed the method for the elaboration of systematic reviews recommended by the Preferred Reporting Items for Systematic Reviews and Meta-Analyses (PRISMA) statement [6]. Although the PRISMA statement is mainly used in systematic reviews of intervention studies, several domains are also applicable to systematic reviews of prevalence [7]. As PRISMA is the most widely used tool for the reporting of systematic reviews, we used it in the present work. The PRISMA checklist for this study is presented in Additional file 1: Material S1.

### Selection criteria and search strategy

We included studies with patients admitted to a hospital setting with suspected lower respiratory tract infection (LRTI) and with SARS-CoV-2 infection confirmed by PCR. Due to the high number of publications, we only included original studies with at least 10 participants and which provided enough information to appraise the methods used. Randomised and non-randomised studies that presented at least one of the following outcomes of interest were included: (a) prevalence of bacterial co-infection in patients with confirmed SARS-CoV-2 infection; (b) the proportion of patients with confirmed SARS-CoV-2 infection that were commenced on empirical antibiotic treatment. Where available, we collected information on the duration and type of antibiotics and on any related adverse events. In cases receiving specific treatment for COVID-19 as part of a clinical trial, we only included standard-of-care comparator arms. We excluded antibiotic use for indications other than bacterial LRTI (e.g., azithromycin used as specific therapy for SARS-CoV-2 at the beginning of the pandemic was excluded). In order for our findings to be readily generalisable, we excluded pregnant women and patients with chronic immunosuppressive conditions, these being specific populations with different and increased infection risk profiles. We also excluded studies that mentioned bacterial co-infection rates but did not provide clinical details (e.g., cost-effectiveness analyses or modelling studies). Given that many authors provided only limited descriptions of antibiotic use, we performed two sub-analyses: one of studies clearly stating bacterial co-infection confirmed by cultures taken less than 48 h from point of admission, and another including only studies that clearly stated the empirical use of antibiotics. In the latter, we also describe any antibiotic stewardship strategies.

We also performed sub-group analyses of any available data on critically ill patients, defined as those patients identified by study authors as requiring admission to high-dependency or intensive care. Definitions of bacterial co-infection provided by study authors were accepted.

We searched the following databases up to May 5, 2021: Pubmed, LILACS, Embase, Web of Science and Cochrane Library. Our search strategy is given in Additional file 1: Material S2. Searches were limited to papers written in English, German, Russian, French, Spanish, or Portuguese. Reference lists from all included articles were also scrutinised to identify additional studies of potential interest.

### Screening and data extraction

We used a two-stage screening process to identify publications that would be eligible for inclusion: title and abstract, followed by full text review. Any original manuscripts referenced by systematic reviews but not identified by the initial search were also included if they were eligible. All publications were then screened in duplicate and independently by reviewers working in pairs (MC, GG, AG, LK, AM, DC); any disagreements in screening were resolved by a third, independent reviewer (EH or BP). Data from eligible papers were extracted by two independent reviewers into separate, piloted and standardised Microsoft Excel spreadsheets; the third reviewer was then asked to resolve any discrepancies and a single consensus dataset was produced after discussion.

### Data analysis

We present the results of all included studies according to the selected outcomes of interest. We analysed our data using a proportion meta-analysis. We applied an arc-sine transformation to stabilise the variance of proportions (Freeman-Tukey variant of the arc-sine square-root of transformed proportions method), where y = arcsine[√(r/(n + 1))] + arcsine[√(r/(n + 1)/(n + 1)], with a variance of 1/(n + 1), with n being the population size. The pooled proportion was calculated as the back-transformation of the weighted mean of the transformed proportions, using inverse arcsine variance weights for the fixed and random effects models. Where heterogeneity between studies was found we applied DerSimonian-Laird weights for the random effects model. We calculated the I^2^ statistic as a measure of the overall variation in the proportion that was attributable to between-study heterogeneity. STATA 17.0 was used for all analyses.

### Study quality assessment

To describe the quality of the prevalence data extracted from the included studies, we used The Joanna Briggs Institute (JBI) Critical Appraisal Checklist for cross-sectional/prevalence data [8]. This is a tool that has been developed acknowledging that prevalence data can come from different study designs, as in our case.

Quality of included studies were assessed independently by two investigators; any disagreements were resolved by a third senior investigator.

## Results

**Fig. 1:**
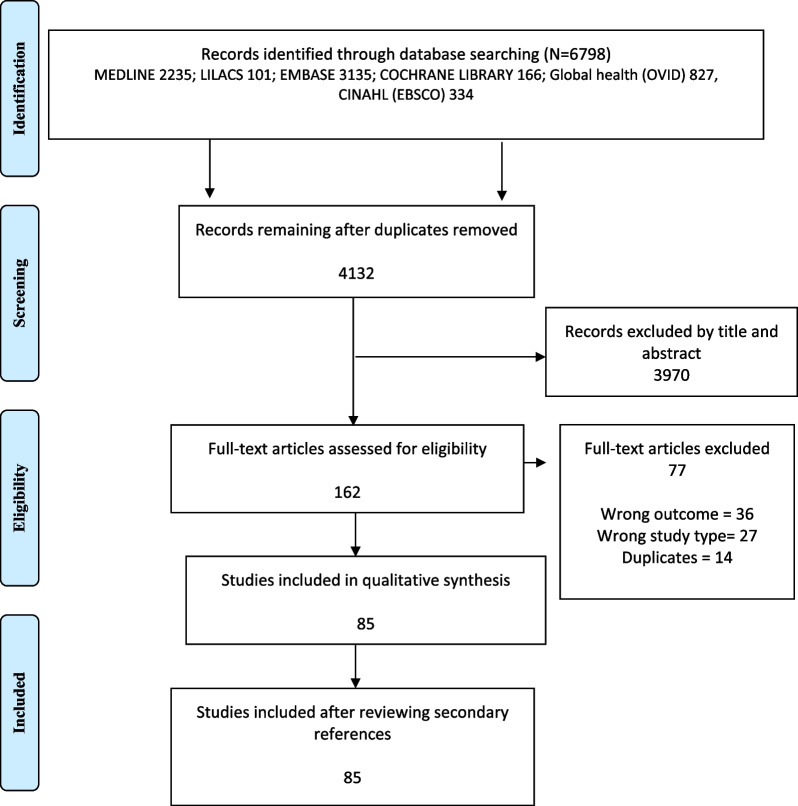
Flowchart of included studies. Moher D, Liberati A, Tetzlaff J, Altman DG, The PRISMA Group (2009). Preferred Reporting Items for Systematic Reviews and Meta-Analyses: The PRISMA Statement. PLoS Med 6(7): e1000097. https://doi.org/10.1371/journal.pmed1000097. For more information, visit www.prisma-statement.org

Database searches identified 6798 studies. After removing duplicates and reviewing the secondary reference lists from included papers we screened a total of 4,132 studies for title and abstract. Of these, 162 (3.9%) went to full text review and 85 (2.1%) were selected for data extraction (Fig. 1).

The independent assessment of the quality of included papers is described in the Additional file 1: Material S3. Using the selected quality assessment tool, we identified that the majority of included studies had appropriate samples for their specific objectives, adequate description of participants and diagnosis of condition.

Data derived from a total of 31,123 individuals were included for analysis. The study designs of all included papers comprised case series, cohorts, registries, and clinical trials. The majority of papers were from China (29, 34.1%) and USA (16, 18.8%). The main characteristics of the included studies are shown in Table 1. Full references of included studies are provided in Additional file 1: Material S4.

**Table 1:** Main characteristics of included studies

Author, Year (Ref)	Country	Centre(s)	Study period	N	Age (years) Mean/SD(range) or *Median/IQR	Female (%)	Ventilatory support		ITU admission	Mortality (%)
							NIV	MV		
Alharty et al., 2020 (1)	SaudiArabia	Single	20/03/20–31/05/20	352	50.6/13.3	45/352 (12.8%)	0/352 (0%)	352/352 (100%)	352/352 (100%)	113/352 (32.1%)
Allou et al., 2021 (2)	France	Single	01/03/20–30/04/20	36	66/56–77*	11/36 (30.5%)	3/36 (8.3%)	2/36(5.6%)	10/36 (27.8%)	0/36 (0%)
Amit et al., 2020 (3)	Israel	Multi	05/03/20–27/04/20	156	72/60–82*	48/156 (30.8%)	39/156 (25%)	93/156(59.7%)	156/156 (100%)	87/156 (55.8%)
Asmarawati et al., 2021 (4)	Indonesia	Single	14/03/20–30/09/20	218	52.4/14.4	98/218 (44.9%)	NR	23/218 (10.5%)	52/218 (23.8%)	21/218 (9.6%)
Ayding Bahat et al., 2020 (5)	Turkey	Single	11/03/20–24/04/20	25	60.5/15	15/25 (60%)	NR	NR	8/25(32%)	5/25(20%)
Balena et al., 2020 (6)	Italy	Single	01/03/20–15/06/20	148	80/72–86*	83/148 (66%)	31/148 (21%)	4/148 (4%)	NR	34/128 (23%)
Baraboutis et al., 2020 (7)	Greece	Single	16/03/20–12/04/20	49	63/20–95*	19/49 (38.8%)	NR	8/49 (16.3%)	NR	6/49 (12.2%)
Bardi et al., 2021 (8)	Spain	Single	01/03/20–01/06/20	140	61/57–67*	32/140 (23%)	NR	134/140 (96%)	140/140 (100%)	51/140 (36%)
Barrasa et al., 2020 (9)	Spain	Single	04/03/20–31/03/20	48	67/53–74*	21/48 (43.8%)	0/48 (0%)	45/48 (94%)	48/48(100%)	16/48 (36%)
Barry et al., 2020 (10)	Saudi	Single	22/03/20–31/05/20	99	44.0/(19–87)	33/99 (33.3%)	9/99 (9.1%)	13/99 (13.1%)	22/99(22.2%)	12/99 (12%)
Basakaran et al., 2021 (11)	UK	Multi	21/02/20–01/05/20	254	59/49–69*	90/254 (35.4%)	NR	151/254 (59.5%)	254/254 (100%)	2/254 (0.8%)
Bhatt et al., 2021 (12)	USA	Multi	01/03/20–07/05/20	375	63.2/16.2	146/375 (38.9%)	7/375 (1.8%)	17/375 (4.5%)	175/375 (46.7%)	149/375 (39.7%)
Buckner et al., 2020 (13)	USA	Multi	02/03/20–26/03/20	105	69/23–97*	52/105 (49.5%)	1/105 (1.0%)	17/105 (16.2%)	51/105 (48.6%)	35/105(33.3%)
Chen et al., 2020 (14)	China	Single	01/01/20–10/02/20	203	54/(20–91)	95/203 (46.8%)	NR	39/203 (19.2%)	39/203 (19.2%)	26/203 (12.8%)
Chen et al., 2021 (15)	China	Single	11/01/20–31/03/20	408	48/34–60*	212/408 (51.9%)	NR	NR	NR	3/408 (0.7%)
Cheng et al., 2020 (16)	China	Single	08/01/20–08/05/20	147	36/24–54*	62/147 (42.1%)	NR	NR	3/147 (2%)	0/147 (0%)
Chengy et al., 2020 (17)	China	Single	01/01/20–18/03/20	62	61/49.3–67.5*	44/62 (70.9%)	NR	NR	NR	0/62 (0%)
Chong et al., 2021 (18)	USA	Single	8/03/20–22/06/20	244	63/51–75*	96/244 (40.6%)	18/244 (7.4%)	71/244 (29.1%)	118/244 (48.4%)	NR
Choubey et al., 2021 (19)	UK	Single	01/03/20–31/05/20	209	NR	NR	NR	NR	NR	NR
Contou et al., 2020 (20)	France	Single	01/03/20–30/02/20	92	NR/(55–70)	19/92 (20.6%)	NR	83/92 (90%)	92/92 (100%)	45/92 (49%)
D’Onofrio et al., 2020 (21)	Belgium	Single	12/03/20–12/04/20	110	73/60–82*	62/110 (56.4%)	NE	NE	29/110(26.4%)	34/110 (30.9%)
Desai et al., 2020 (22)	Italy	Single	01/04/20–30/09/20	536	62.9/12.8	5268/536 (50%)	NR	NR	NR	116/536 (21.6%)
Dolci et al., 2020 (23)	Italy	Single	01/02/20–31/03/20	83	61/(49–67)	11/83 (13.3%)	NR	NR	NR	44/83 (53.0%)
Ekadashi et al., 2021 (24)	India	Single	23/03/20–23/08/20	158	NR	NR	NR	NR	NR	NR
Elabbadi et al., 2021 (25)	France	Single	01/02/20–31/05/20	101	61/53–69*	22/101 (10.8%)	NR	83/101(82.2%)	101/101 (100%)	21/101 (20.8%)
Falcone et al., 2021 (26)	Italy	Single	04/03/20–30/04/20	315	NR	105/315 (33.3%)	68/315 (21.6%)	55/315 (17.5%)	85/315 (26.9%)	70/315 (22.2%)
Fan et al., 2021 (27)	China	Single	01/01/20–31/01/20	55	46.46/14.41	25/55 (45.5%)	NR	NR	NR	0/55 (0%)
Garcia-Vidal et al., 2021 (28)	Spain	Single	01/02/20–30/04/20	989	62/48–74*	437/989 (44.2%)	NR	NR	146/989 (14.8%)	99/989 (10.0%)
Gayam et al., 2020 (29)	USA	Single	01/03/20–30/04/20	350	NR	NR	NR	NR	NR	NR
Goncalves et al., 2021 (30)	USA	Single	01/03/20–20/04/20	242	66/14.75	119/242 (49.2%)	NR	NR	NR	52/242 (22.7%)
Guan et al., 2020 (31)	China	Single	NR	61	56.8/15.1	NR	NR	NR	NR	1/61 (1.6%)
He et al., 2020 (32)	China	Single	01/02/20–28/02/20	192	45/NR	93/192(48.4%)	NR	NR	NR	5/192 (2.6%)
He et al., 2021 (33)	China	Multi	01/01/20–28/02/20	905	47/35–57*	463/905 (51.2%)	NR	NR	NR	57/905 (6.2%)
Huang et al., 2020(34)	China	Single	16/12/19–02/01/20	41	49.0/41.0–58.0*	11/41 (26.8%)	10/41 (24%)	2/41(5%)	NR	6/41(14.6%)
Huang et al., 2020 (34)	China	Single	16/12/19–02/01/20	13	49/41–61*	2/13 (15%)	8/13 (62%)	2/13 (15%)	13/13 (100%)	5/13(38%)
Huang et al., 2021 (35)	USA	Single	01/03/20–31/05/20	41	66.6/19.1	21/41(51.2%)	26/41(63.4%)	15/41(37%)	15/41(36.6%)	15/41(36.6%)
Hughes et al., 2020 (36)	UK	Multi	20/02/20–20/04/20	836	69/55–81*	317/836 (37.9%)	NR	NR	NR	NR
Humières et al., 2021(37)	France	Multi	29/01/20–31/05/20	197	59/(50–68)	48/197 (24.8%)	NR	129/197(67%)	197/197 (100%)	71/197 (26.0%)
Karaba et al., 2021 (38)	USA	Multi	01/03/20–31/05/20	1016	62/48–74*	473/1016(46.5%)	NR	NR	NR	NR
Karami et al., 2021 (39)	Netherlands	Multi	01/03/20–31/05/20	925	70/59–77*	334/925 (36.1%)	NR	NR	NR	214/925 (23.1%)
Kimming et al., 2020(40)	USA	Single	01/03/20–27/04/20	111	NR	49/111 (44.1%)	NR	NR	NR	30/111 (27%)
Kolenda et al., 2020 (41)	France	Multi	01/03/20–15/04/20	99	NR	NR	NR	NR	NR	NR
Lardaro et al., 2021 (42)	USA	Multi	01/03/20–30/04/20	542	64.8/16.5	273/542 (50.4%)	NR	162/542 (29.9%)	86/542 (15.9%)	78/542 (14.4%)
Li et al., 2020 (43)	China	Single	20/01/20–14/02/20	225	50.0/14.0	105/225 (46.7%)	NR	NR	NR	2/225 (0.9%)
Li et al., 2020 (44)	China	Single	27/01/20–17/03/20	1495	NR	NR	NR	NR	NR	NR
Liu et al., 2020 (45)	China	Single	18/01/20–12/03/20	140	65.5/54.3–73.0*	91/140 (65.0%)	NR	NR	NR	NR
Liu et al., 2021 (46)	China	Single	01/01/20–28/02/20	53	38/28–47*	27/53 (50.9%)	32/53 (63.4%)	1/53(1.9%)	1/53 (1.9%)	0/53 (0%)
Liu et al., 2021 (47)	China	Single	26/01/20–18/03/20	1123	61/50–69*	563/1123 (50.1%)	NR	NR	NR	111/1123 (9.9%)
Mady et al., 2020 (48)	Saudi Arabia	Single	12/08/20–12/09/20	61	51/42.5–58.8*	7/61 (11.5%)	32/61 (52.5%)	29/61 (47.5%)	61/61 (100%)	19/61 (31.1%)
Mahmoudi et al., 2020 (49)	Iran	Single	17/02/20–20/10/20	340	NR	NR	NR	NR	NR	NR
Mason et al., 2021 (50)	UK	Multi	01/03/20–31/05/20	800	NR/(18–100)	310/800 (38.7%)	NR	NR	NR	NR
M. Movahed et al., 2021 (51)	Iran	Single	22/02/20–19/04/20	854	55.6/17.63	382/854 (44.7%)	NR	NR	183/854(21/4%)	119/854 (13.9%)
Nassir et al., 2021 (52)	Pakistan	Single	01/02/20–30/06/20	100	58/49–57*	11/100 (11%)	NR	35/100 (35%)	79/100 (79%)	30/100 (30%)
Nebreda et al., 2020 (53)	Spain	Single	08/03/20–31/05/20	712	NR	NR	NR	NR	NR	NR
Pulia et al., 2021 (54)	USA	Multi	15/03/20–18/05/20	73	NR	38/73 (52.1%)	NR	8/73 (10.9%)	NR	NR
Quartuccio et al., 2020(55)	Italy	Single	01/02/20–30/04/20	69	56.2/14.2	25/69 (36.2%)	0/69 (0%)	0/69 (0%)	0/69 (0%)	0/69 (0%)
Richardson et al., 2020 (56)	USAe	Multi	01/03/20–04/04/20	5700	63.0/52.0–75.0	2263/5700 (39.7%)	NR	320/5700 (5.6%)	373/5700 (6.5%)	553/5700 (9.7%)
Rippa et al., 2021 (57)	Italy	Single	25/02/20– 6/04/20	731	64/(55–76)	235/731 (32.1%)	NR	NR	45/731(6.1%)	NR
Rothe et al. 2020 (58)	Germany	Single	01/02/20–30/04/20	140	63.5/(17–99)	50/140 (35.7%)	NR	41/14 0(29.3%)	56/140 (40%)	18/140 (12.8%)
Seaton et al., 2020 (59)	Scotland	Multi	20/04/20–30/04/20	531	72/61–82*	257/531 (48.4%)	NR	NR	110/531 (20.7%)	NR
Shah et al., 2020 (60)	USA	Single	03/02/20–31/03/20	33	63/50–75*	12/33 (36.4%)	0/33(0%)	6/11 (55%)	11/26 (42%)	1/26 (4%)
Shao et al., 2020 (61)	China	Multi	23/01/20–23/03/20	126	NR/(19–91)	58/126 (46.0%)	NR	NR	NR	1/126 (0.1%)
Sharifipour et al., 2020 (62)	Iran	Multi	NR	19	67/4.6	8/19 (42.1%)	NR	NR	19/19(100%)	18/19(94.7%)
Silva et al., 2021 (63)	Brazil	Single	01/05/20–30/11/20	212	NR	86/212 (40.5%)	NR	NR	212/212(100%)	107/212(52.9%)
Singh et al., 2021 (64)	USA	Single	16/03/20–01/08/20	4259	45.2/20–43*	2513/4259 (55.5%)	NR	NR	NR	NR
Soogard et al., 2021 (65)	Switzerland	Single	25/02/20–31/05/20	162	64.4/50.4–74.2*	63/162(38.9%)	NR	34/162(20.9%)	41/162(25.3%)	17/162(10.5%)
Staub et al., 2021 (66)	USA	Single	01/03/20–15/05/20	131	56/17.4	53/131(39.7%)	NR	NR	NR	13/131 (9.9%)
Stevens et al., 2021 (67)	USA	Single	01/03/20–28/04/20	346	45/18	176/346 (51%)	NR	NR	0/346 (0%)	0/346 (0%)
Tang et al., 2021 (68)	China	Single	28/01/20–15/03/20	78	47.7/17.2	37/78(47.4%)	NR	NR	8/78 (10.3%)	NR
Thelen et al., 2021 (69)	Netherlands	Multi	28/02/20–02/06/20	678	70/58–78*	235/678 (34.7%)	NR	NR	6/678 (0.9%)	191/678 (28.3%)
Townsend et al., 2020 (70)	Ireland	Multi	01/03/20–31/04/20	117	NR	43/117 (36.8%)	NR	NR	34/117 (29.1%)	17/117 (14.5%)
Vanhomwegen et al., 2021 (71)	Belgium	Single	03/03/20–02/05/20	66	61/49–71*	25/66 (38%)	NR	NR	66/66 (100%)	20/66 (30.3%)
Vaughn et al., 2021 (72)	USA	Multi	01/03/20–01/06/20	1705	64.7/53.0–76.7*	820/1705 (48.1%)	13/1705 (0.8%)	116 /1705 (6.8%)	NR	325/1705 (19.1%)
Wan et al., 2020 (73)	China	Single	23/01/20–08/02/20	135	47.0/36.0–55.0*	63/135 (46/7%)	34/135 (19.4%)	1/135 (0.7%)	40/135 (29.6%)	1/135 (0.7%)
Wan et al., 2020 (73)	China	Single	23/01/20–08/02/20	40	56.0/52.0–73.0*	19/40 (47.5%)	27/40 (67.5%)	1/40 (2.5%)	40/40	1/40 (2.5%)
Wang et al., 2020 (74)	China	Single	29/01/20–10/02/20	28	68.6/9.0 (53–82)	7/28 (25%)	11/28 (39.3%)	7/28 (25%)	14/28 (50%)	12/28 (42.9%)
Wang et al., 2020 (74)	China	Single	29/01/20–10/02/20	14	71.4/7.9	4/14(71.4%)	11/14 (79.6%)	7/14 (50%)	14/14 (100%)	12/14 (85.7%)
Wang et al., 2020 (75)	China	Single	01/01/20–06/02/20	339	69.0/65.0–76.0*	173/339(51.0%)	NR	NR	65/339 (19.2%)	65/339 (19.2%)
Wang et al., 2021 (76)	UK	Multi	01/03/20–30/04/20	1396	67.4/16.2	NR	NR	NR	226/1396 (16.2%)	420/1396 (30.1%)
Xu et al., 2021 (77)	China	Single	Up to 12/03/20	62	56.5/45.3–74.8*	27/62 (44%)	24/62 (45%)	15/62 (24%)	62/62 (100%)	7/62 (11.3%)
Yang et al., 2020 (78)	China	Single	05/01/20–22/02/20	251	NR	128/251 (51.0%)	NR	NR	NR	21/251 (8.4%)
Zhang et al., 2020 (79)	China	Single	02/01/20–10/02/20	221	55.0/39.0–66.5*	113/221 (51.1%)	26/221 (12.2%)	16/221 (7.2%)	NR	12/221 (5.4%)
Zhang et al., 2020 (80)	China	Single	16/01/20–03/02/20	140	57.0/25.0–87.0*	69/140 (49.3%)	NR	NR	NR	NR
Zhang et al., 2020 (81)	China	Single	22/01/20–30/04/20	38	64.7/13.7	6/38 (15.8%)	NR	NR	NR	NR
Zhang et al., 2020 (82)	China	Single	10/12/19–20/02/20	134	60.8/12.9	47/134 (35.1%)	91/134 (67.9%)	79/134 (58.9%)	134/134 (100%)	101/134 (75.4%)
Zhang et al., 2021 (83)	China	Single	01/01/20–28/02/20	91	74.9/68–82*	52/91 (57.1%)	3/91 (3.3%)	11/91 (12.1%)	NR	5/91 (55.6%)
Zhang et al., 2020 (84)	China	Single	01/01/20–31/03/20	365	46.8/15.5	189/365(51.8%)	NR	NR	NR	2/365(0.5%)
Zhao et al., 2020 (85)	China	Single	01/01/20–28/02/20	1000	61/46–70*	534/1000 (53.4%)	147/1000 (14.7%)	43/1000 (4.3%)	63/1000 (6.3%)	119/1000 (11.9%)

N: number. NR: not reported. IQR: interquartile range. SD: standard deviation, SOCa: standard of care

NIV: Non-invasive ventilation, MV: mechanical ventilation, ITU: intensive treatment unit

Full list of references are in Additional file 1: Material S4

### Bacterial co-infection prevalence

**Fig. 2:**
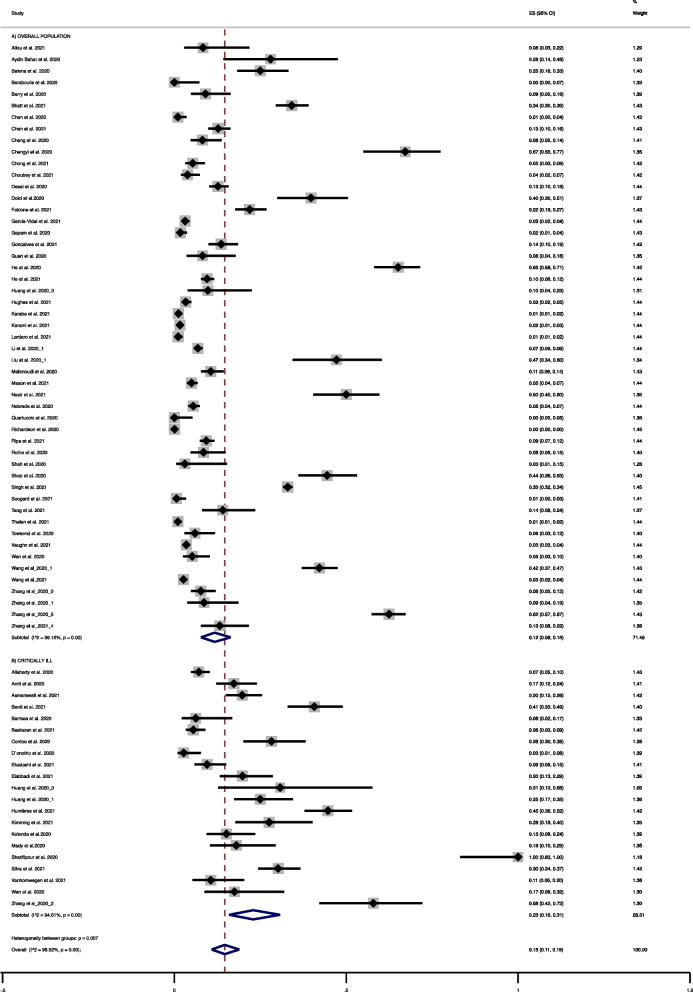
Meta-analysis of bacterial prevalence in patients with SARS-Cov-2: **a** overall population, **b** critically ill patients

We included 70 studies that reported on the prevalence of bacterial co-infection (including critically ill patients and not critically ill patients) (Table 2). Meta-analysis of these studies showed an overall prevalence of bacterial co-infection of 12% (51 studies, 95% CI 8% to 16%; I^2^ 99.2%) (Fig. 2A); subgroup meta-analysis of critically ill patients showed a prevalence of 23% (21 studies, 95% CI 16 to 31%; I^2^ 94.6%) (Fig. 2B).

**Table 2:** Studies reporting bacterial co-infection in patients with COVID-19

Study	n	N	Patient group	Definition of bacterial co-infection	Microorganism identified
Alharty et al., 2020 (1)	25	352	Critically ill	Nosocomial acquired bacterial infection by culture (15 VAP + 10 CLI)	Most common: *Acinetobacter baumannii,* and MRSA
Allou et al., 202 1(2)	3	36	Not specific: General including critically ill	Co-infections. Method: Measured by multiplex PCR, pneumococcal and Legionella urinary antigen tests, cytobacteriological examination of sputum cultures, and serology of atypical respiratory pathogens	*Branhamella catarrhalis* = 1 *Streptococcus pneumoniae* and *Haemophilus influenza* = 1 MSSA = 1
Amit et al., 2020 (3)	27	156	Critically ill	Secondary infection. Method: NR	NR
Asmarawati et al., 2021 (4)	43	218	Moderate to Critically ill	Expressed as co-infection and secondary infections Method: Blood and sputum and urine cultures	Most common presented Blood culture: ESBL-producing *Klebsiella pneumoniae*, *Pseudomonas* spp. Sputum: *Acinetobacter baumannii*, *Klebsiella pneumoniae*
Ayding Bahat et al., 2020 (5)	7	25	Haemodialysis	Secondary infection. Method: NR	NR
Balena et al., 2020 (6)	32	128	Elderly	At least one secondary infection by authors. Method: NR	NR
Baraboutis et al., 2020 (7)	0	49	Overall	Blood and sputum cultures, urine pneumococcal and legionella antigen	NR
Bardi et al., 2021 (8)	57	140	Not specific: General	Nosocomial infection (30 LRTI, 21 VAP, 28 BSI, 24 CRBSI, 7 UTI, 2 soft tissue infections) Method: Cultures	Most common presented: BSI: *Enterococcus faecium* (43%), followed by *Enterococcus faecalis* (21%) CRBSI: coagulase-negative staphylococci (54%), *Enterococus faecium* (17%) VAP: *Staphylococcus aureus* (24%)
Barrasa et al., 2020 (9)	3	48	Critically ill	NR	NR
Barry et al., 2020 (10)	9	99	Not specific: General including critically ill	Method: Sputum culture and blood culture	Sputum culture: *Stenotrophomonas maltophilia* = 1*, Klebsiella pneumoniae* = 1 Blood culture*: Staphylococcus epidermidis* = 4*; Enterococcus faecalis* = 1*; Corynebacterium amycolatum* = 1; *Bacillus pumilus* = 1
Basakaran et al., 2021 (11)*	14	254	Critically ill	Method: Standard culture (blood, sputum, tracheal-aspirate, bronchoalveolar lavage, urine) and validated culture-independent tests such as respiratory viral PCR and urinary antigens	The most common potential co-pathogens identified were Gram negative bacteria, including *Klebsiella* spp. (23) and *Escherichia coli* (20)
Bhatt et al., 2021 (12)	128	375	Not specific: General including critically ill	Method: Blood culture	Most common: *Staphylococcus epidermidis,* MSSA *Enterococcus faecalis, Escherichia coli,* MRSA
Chen et al., 2020 (14)	2	203	Elderly	Method: PCR	*Mycoplasma pneumoniae* = 2
Chen et al., 2021 (15)*	25	408	Not specific: General	Method: Blood culture, respiratory culture, serology, PCR and metagenomic next-generation sequencing	*Mycoplasma pneumoniae* = 3 *Haemophilus influenzae* = 6 *Klebsiella pneumoniae* = 2 *Streptococcus pneumoniae* = 1 *Staphylococcus aureus* + *Streptococcus pneumoniae* = 1 *Staphilococcus aureus* + Haemophilus influenzae = 1 MRSA + *Haemophilis influenzae* + *Streptococcus pneumoniae* = 1
Cheng et al., 2020 (16)*	12	147	Not specific: General including critically ill	Method: Blood culture, respiratory culture, serology, PCR	MSSA + *Haemophilus influenzae* = 1, MSSA = 8, *Pseudomonas aeruginosa* = 1, *Haemophilus influenza* = 2
Chengy et al., 2020 (17)	43	64	Not specific: General	Method: Positive culture or clinical/laboratory suspicion	NR
Chong et al., 2021 (18)*	13	244	Not specific: General including critically ill	Method: Respiratory tract cultures ± concurrent positive blood culture	*Haemophilus influenzae* = 3*, Klebsiella penumoniae* = 3 *Pseudomona aeruginosa* + MSSA = 3*, Corynebacterium striatum* = 2 MRSA = 2 Others = *Citrobacter freundii, Moraxella catarrhalis, Enterobacter aerogenes, Klebsiella aerogenes* (respiratory culture)
Choubey et al., 2021 (19)	8	209	Not specific: General	Method: Mycoplasma pneumoniae serology	*Mycoplasma pneumoniae* = 8
Contou et al., 2020 (20)*	26	92	Critically ill	Method: Cultures, PCR, antigen	Most common: MSSA (10/32, 31%), *Haemophilus influenzae* (7/32, 22%), *Streptococcus pneumoniae* (6/32, 19%), *Enterobacteriaceae* spp. (5/32, 16%)
D’Onofrio et al., 2020 (21)*	3	10	Not specific: General including critically ill	Method: Cultures, PCR, antigen	*Staphylococus hominis* = 1, *Corynebacterium aurimucosum* = 1, *Streptococcus pyogenes* = 1
Desai et al., 2020 (22)	68	536	Not specific: General	Method: *Streptococcus pneumoniae* urinary antigen (u-Ag)	*Streptococcus pneumoniae* = 68
Dolci et al., 2020 (23)	33	83	Not specific: General	Definition/Method: positivity of blood cultures and/or of cultures of lower respiratory tract specimens (bronchoalveolar lavage fluid or bronchial aspirate)	NR
Ekadashi et al., 2021 (24)	15	158	Not specific: General including critically ill	Blood culture	Coagulase negative *Staphylococcus* spp*.* (11, 73.3%)
Elabbadi et al., 2021 (25)*	20	101	Critically ill	Method: Culture (respiratory, blood), urinary antigen	Gram positive = 11, Gram negative = 13
Falcone et al., 2021 (26)*	69	315	Not specific: General including critically ill	Definition: Hospital acquired > 48 h Method: Blood culture	Enterobacterales (44.9%), non-fermenting Gram negative bacilli (15.6%), Gram positive bacteria (15.6%)**
Garcia-Vidal., 2021 (28)*	21	989	Not specific: General including critically ill	Method: Culture (respiratory, blood), urinary antigen. < 24 h	*Streptococcus pneumoniae* + *Moraxella catarrhalis* = 1 *Staphylococcus aureus* + *Haemophilus influenzae* = 1
Gayam et al., 2020 (29)*	6	350	Not specific: General	Method: Mycoplasma PCR	*Mycoplasma pneumoniae* = 6
Goncalves et al., 2021 (30)*	46	242	Not specific: General	Definition/Method: Clinical features and positive blood, sputum, urine, or tissue culture results	NR
Guan et al., 2020 (31)	5	61	Not specific: General	Method: Blood and respiratory culture	Gram negative bacteria = *2* Gram negative + Gram positive bacteria = 3
He et al., 2020 (32)	125	192	Not specific: General	Method: PCR	*Streptococcus pneumoniae* = 14, *Bordetella pertussis* = 19*, Streptococcus.pyogenes* = 3, *Staphylococcus aureus* = 1*, Mycobacterium tuberculosis* = 7*, Neisseria meningitidis* = 7, *Haemophilus influenzae* = 17*, Pseudomonas aeruginosa* = 57
He et al., 2021 (33)*	86	905	Not specific: General	Definition/Method: Clinical diagnosis based on clinical findings combined with laboratory and radiology findings	NR
Huang et al., 2020 (34)	4	41	Not specific: General including critically ill	Definition/Method: Positive culture of a new pathogen from a lower respiratory tract specimen	NR
Huang et al., 2020 (34)	4	13	Critically illl	Definition/Method: Positive culture of a new pathogen from a lower respiratory tract specimen	NR
Huang et al., 2021 (35)	7	41	Critically illl	Method: Culture NB: Majority of infections were considered nosocomial	NR
Hughes et al., 2020 (36)	21	643	Not specific: General	Method: Blood culture, respiratory culture, pneumococcal antigen, *Legionella* antigen	CRBSI: *Klebsiella pneumoniae* = 1, VAP: *Enterobacter cloacae* = 1 CLI: *Enterococcus* spp. = 2, *Pseudomonas aeruginosa* = 1
Humières et al., 2021 (37)	88	197	Critically ill	Definition/Method: Nosocomial infections. Clinical features and positive blood, sputum, urine, or tissue culture results	NR
Karaba et al., 2021 (38)*	12	1016	Not specific: General	Definition/Method: Clinical, laboratory, and radiographic criteria plus microbiologic diagnosis	Only confirmed: Sputum culture: MSSA = 1
Karami et al., 2021 (39)*	12	925	Not specific: General	Method: Respiratory cultures, pneumococcal antigen, *Legionella* antigen	Most common: *Staphylococcus.aureus, Escherichia coli, Stenotrophomonas maltophilia*
Kimming et al., 2020 (40)	16	58	Critically ill. Soc	Definition: Including hospital acquired infections Method: Cultures	NR
Kolenda et al., 2020 (41)	15	99	Critically ill	Method: PCR and culture	Most common: *Staphylococcus.aureus,*and *Haemophilus influenzae*
Lardaro et al., 2021 (42)*	6	542	Not specific: General including critically ill	Method: Blood cultures	NR
Li et al., 2020 (a) (44)	102	1495	Not specific: General	Method: Cultures	*Acinetobacter baumannii *(57/159, 35.8%), *Klebsiella. pneumoniae (49/159, 30.8%,), Stenotrophomonas maltophilia (10/159, 6.3%)*
Mady et al., 2020 (48)	11	61	Critically ill	Definition: Including hospital acquired infections Method: Blood and respiratory cultures	Blood culture: *Staphylococcus aureus* = NR, Vancomycin resistant enterococcus (sensitive to tigecycline) = NR*, Acinetobacter baumannii* = NR VAP: *Pseudomonas* spp. = 3*, Acinetobacter baumannii* = 3
Mahmoudi et al., 2020 (49)	36	340	Not specific: General	Method: Endotracheal and blood cultures	*Klebsiella* spp*.* (11, 25.59%), MSSA (9, 20.93%), *Escherichia.coli* (7, 16.28%), MRSA (6, 13.95%), *Enterobacter* spp. (5, 11.63%), *Streptococcus pneumoniae* (1, 2.32%), *Pseudomonas* aeruginosa (4, 9.30%)
Mason et al., 2021 (50)	40	800	Not specific: General	Method: Sputum, blood, urine ag, Mycoplasma PCR	NR
Nassir et al., 2021 (52)	50	100	Not specific: General including critically ill	Method: Blood and respiratory cultures	NR
Nebreda et al., 2020 (53)*	39*	712	Not specific: General including critically ill	Method: Blood and respiratory culture	Most common: Gram negative bacilli (59%), *Escherichia.coli* (47%) *Enterococcus faecalis* (21%)*, Streptococcus pneumoniae* (33%) and *Staphylococcus aureus* (33%)
Quartuccio et al., 2020 (55)	0	69	SOCa	Method: Respiratory and blood cultures	NR
Richardson et al., 2020 (56)	3	5700	Not specific: General including critically ill	Method: PCR for extensive respiratory panel including atypical bacteria	*Chlamydia pneumoniae* = 2 *Mycoplasma pneumoniae* = 1
Rippa et al., 2021 (57)	68	731	Not specific: General including critically ill	Definition: Clearly stated as secondary co-infection within > 48 h of admission	BSIs: Gram positive bacteria (76/106, 71.7%), of which 53/76, 69.7% were coagulase-negative staphylococci BSIs: Gram negative bacteria (23/106, 21.7%), of which 7/23, 30.4% *Acinetobacter baumannii,* and 5/23, 21.7% were *Escherichia coli* LRTIs: Gram-negative bacteria (14/26, 53.8%)
Rothe et al., 2020 (58)	10	118	Not specific: General including critically ill	Method: Blood cultures, *Legionella pneumophila* and *Streptococcus pneumoniae* urinary antigens	Only blood cultures were positive (n = 10)
Shah et al., 2020 (60)	1	33	Overall	Method: Blood and respiratory cultures	Blood culture: *Enterococcus faecium* = *1* Respiratory culture: *Stenotrophomonas maltophilia* = 1
Shao et al., 2020 (61)	56	126	Not specific: General including critically ill	NR	NR
Sharifipour et al., 2020 (62)	19	19	Critically ill	Definition: nosocomial infections Method: Cultures	*Acinetobacter baumannii* (17, 90%) *Staphylococcus aureus* (2, 10%)
Silva et al., 2021 (63)	64	212	Critically ill	Method: Cultures	*Staphylococcus *spp*.* (29, 45.3%), *Acinetobacter *spp*.* (21, 32.8%), *Pseudomonas *spp*.* (21, 32.8%), *Stenotrophomonas* spp*.* (9, 14.06%), *Klebsiella *spp*.* (8, 12.5%), *Enterobacter *spp*.* (6, 9.4%)*, Enterococcus* spp*.* (6, 9.4%)*,* and *Escherichia coli *(4, 6%)
Singh et al., 2021 (64)	1413	4259	Overall	Method: Cultures, serology and PCR	*Heamophilus influenzae* (9.27%), *Staphylococcus aureus* (13.17%), *Streptococcus pneumoniae* (1.94%)
Soogard et al., 2021 (65)*	1	162	Not specific: General including critically ill	Definition/Method: Community-acquired bacterial pneumonia was defined as a microbiology-confirmed pneumonia diagnosed concurrent with SARS-CoV-2 infection or within < 48 h of hospital admission	NR
Tang et al., 2021 (68)	5	78	Not specific: General including critically ill	Method: Mycoplasma pneumonia PCR	*Mycoplasma pneumoniae* = 5
Thelen et al., 2021 (69)*	7	678	Not specific: General including critically ill	Method: Blood cultures	*Escherichia coli* = 2, *Klebsiella pneumoniae* = 1*, Pseudomonas aeruginosa* = 1*, Streptococcus pneumoniae* = 2*, Staphylococcus aureus* = 1
Towsend et al., 2020 (70)*	7	117	Not specific: General including critically ill	Method: Cultures and urinary antigen	*Pseudomonas aeruginosa, Escherichia.coli, Klebsiella pneumoniae,. Oxytoca, Klebsiella aerogenes,* MSSA*, Streptococcus pneumoniae*
Vanhomwegen et al., 2021 (71)*	7	66	Critically ill	Method: Respiratory or blood cultures	NR
Vaughn et al., 2021 (72)*	59	1705	Not specific: general	Method: Blood and sputum cultures, urine pneumococcal and legionella antigen, Mycoplasma pneumonia or Chlamydophila pneumonia PCR	NR
Wan et al., 2020 (73)	7	135	Not specific: General including critically ill	NR	NR
Wan et al., 2020 (73)	7	40	Severe and critically ill	NR	NR
Wang et al., 2020 (75)	143	339	Elderly	NR	NR
Wang et al., 2021 (76)*	37	1396	Not specific: General including critically ill	Method: Blood, lower respiratory tract, urine and other cultures	Blood cultures*: Escherichia coli, Klebsiella pneumoniae, Klebsiella variicola, Proteus mirabilis,* MRSA*,* MSSA and* Staphylococcus epidermidis* Respiratory cultures*: Escherichia coli* (ESBL-producing), group A streptococcus, *Haemophilus influenzae, Pseudomonas aeruginosa,* MSSA
Zhang et al., 2020 (79)	17	221	Not specific: General including critically ill	Definition/Method NR. Likely nosocomial	Mons common: *A.baumannii*, *Escherichia coli* = NR *Pseudomonas aeruginosa*, *Enterococcus* = NR
Zhang et al., 2020 (80)	5	58	Not specific: general	Method: IgM	*Mycoplasma pneumoniae* = 5
Zhang et al., 2020 (81)	22	38	Critically ill	Definition: VAP Method: Cultures	Gram negative bacteria (26, 50.00%), Gram positive bacteria (14, 26.92%), virus (6, 11.54%), fungi (4, 7.69%), and others (2, 3.85%)
Zhang et al., 2021 (83)	12	91	Elderly	NR	NR
Zhang et al., 2020 (84)	228	365	Not specific: general	NR	NR

n: number of patients with reported bacterial co-infection. N: total number of patients. NR: not reported. MRSA: methicillin resistant staphylococcus aureus. MSSA: methicillin susceptible staphylococcus aureus. VAP: ventilator associated pneumonia, CLI: central line infection. ESBL: extended spectrum beta-lactamase. LRTI: lower respiratory tract infection. BSI: bloodstream infection. CRBSI: catheter-related bloodstream infection. UTI: urinary tract infection. PCR: polymerase chain reaction. SOCa: standard of care

*Specifically reported as bacterial co-infections detected 48 h after admission

Full list of references are in Additional file 1: Material S4

**Fig. 3:**
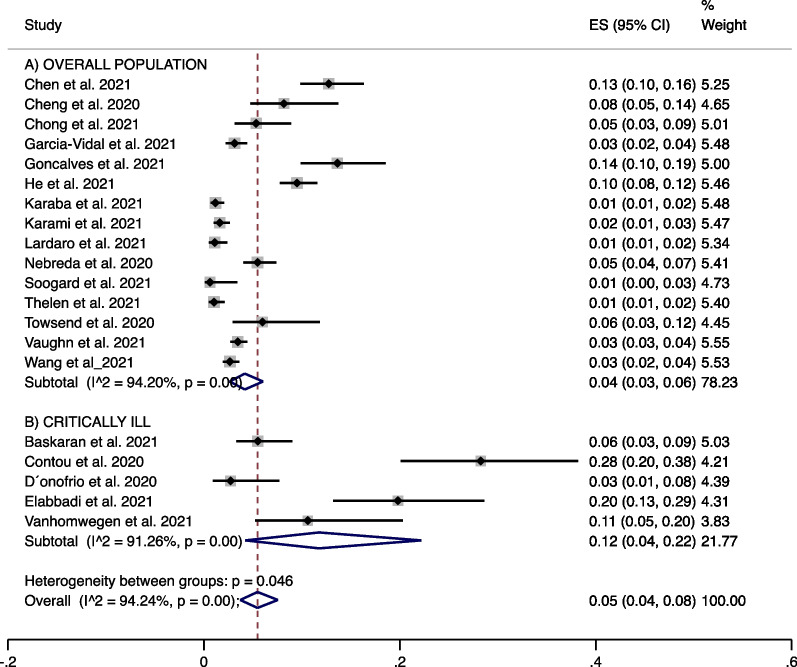
Studies clearly describing bacterial coinfection with microorganism identification in samples taken < 48 h from admission

Twenty studies (31.4%) gave a clear definition of bacterial co-infection, stating that this was diagnosed within 48 h from admission. All of them included cultures, urinary antigen and PCR for definitions of bacterial co-infection. We performed a meta-analysis of this subgroup that showed a prevalence of 4% (15 studies, 95% CI 3% to 6%; I^2^ 94.2%) in the overall population (Fig. 3A) and a bacterial coinfection prevalence of 12% (5 studies, 95% CI 4% to 22%; I^2^ 91.2%) in critically ill patients. (Fig. 3B).

### Antibiotic use

**Fig. 4:**
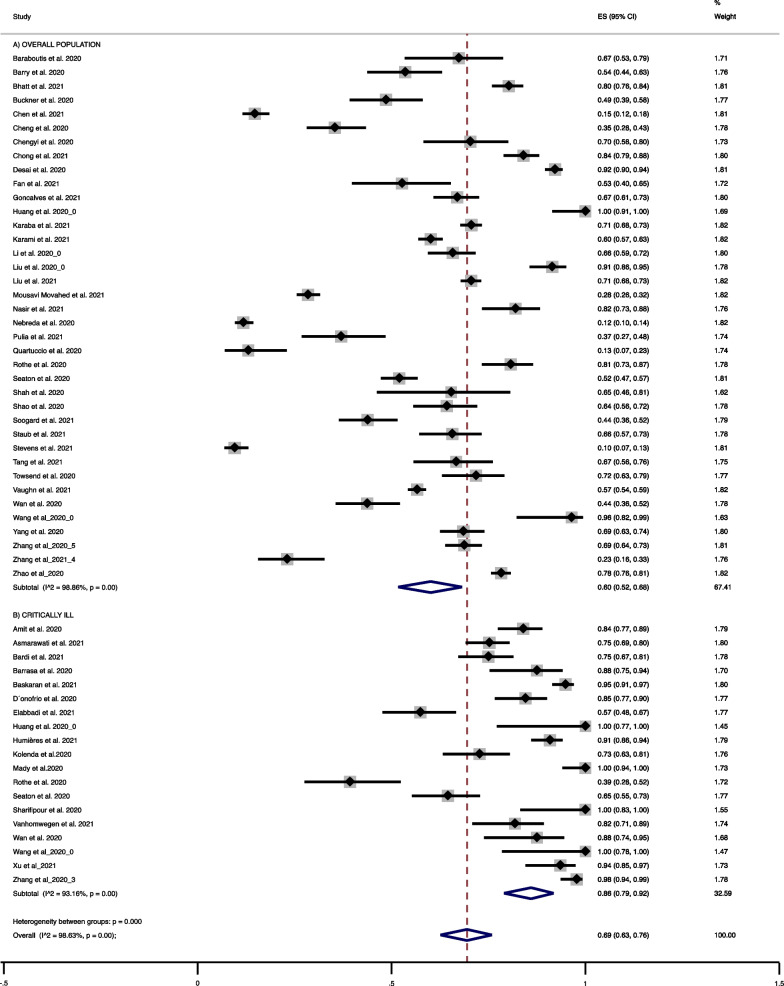
Antibiotic usage in: **a** overall population, **b** critically ill patients

Fifty-two (61.2%) studies were included in the analysis of antibiotic use (Table 3). Meta-analysis showed an overall prevalence of antibiotic use of 60% (38 studies, 95% CI 52% to 76%; I^2^ 98.8%) (Fig. 4A); sub-group analysis restricted to critically ill patients identified a prevalence of antibiotic usage of 86% (19 studies, 95% CI 78% to 92%; I^2^ 93.2%) (Fig. 4B).

**Table 3:** Antibiotic use in patients with COVID-19

Author	n	N	Patient groups	Antibiotics used
Amit et al., 2020 (3)	131	156	Critically ill	NR
Asmarawati et al., 2021 (4)*	164	218	Moderate to critically ill	Quinolones (60.1%), cephalosporins (28.4%), carbapenem (23.8%), and aminoglycosides (5.6%)
Baraboutis et al., 2020 (7)	33	49	Not specific: General	NR
Bardi et al., 2021 (8)	105	140	Critically ill	Ceftriaxone (120, 86%) and/or azithromycin (118, 84%)
Barrasa et al., 2020 (9)	42	48	Critically ill	Ceftriaxone, levofloxacin, beta-lactams, azithromycin, linezolid
Barry et al., 2020 (10)	53	99	Not specific: General including critically ill	NR
Basakaran et al., 2021 (11)	241	254	Critically ill	NR
Bhatt et al., 2021 (12)	301	375	Not specific: General including critically ill	Most common: ceftriaxone, azithromycin, and piperacillin-tazobactam
Buckner et al., 2020 (13)	51	105	Not specific: General including critically ill	NR
Chen et al., 2021 (15)	60	408	Not specific: General	NR
Cheng et al., 2020 (16)*	52	147	Not specific: General	Penicillin & cephalosporins = 46, tetracyclines = 14, quinolones = 3, macrolides = 3
Chengy et al., 2020 (17)	45	64	Not specific: General	NR
Chong et al., 2021 (18)	205	244	Not specific: General including critically ill	NR
D’Onofrio et al., 2020 (21)*	93	110	Not specific: General including critically ill	NR
Desai et al., 2020 (22)	494	536	Not specific: General	Included a combination of ceftriaxone 2 g intramuscular/intravenous twice daily for 7–10 days and azithromycin 500 mg oral once daily for 3 consecutive days. levofloxacin 750 mg oral/intravenous once daily for 5 days was administered when contraindication
Elabbadi et al., 2021 (25)*	58	101	Critically ill	NR
Fan et al., 2021 (27)	29	55	Not specific: General	Moxifloxacin (19/29, 65.52%), Linezolid (3/29, 10.34%)
Goncalves et al., 2021 (30)*	162	242	Not specific: General	NR
Huang et al., 2020 (34)	41	41	Not specific: General including critically ill	NR
Huang et al., 2020 (34)	13	13	Critically ill	NR
Hunieres et al., 2021 (37)	88	197	Critically ill	NR
Karaba et al., 2021 (38)*	717	1016	Not specific: General	NR
Karami et al., 2021 (39)*	556	925	Not specific: General	Amoxicillin/benzylpenicillin (34, 6.1%), Ceftriaxone (95, 17.1%), Cefuroxime (350, 62.9%) Other antibiotics (48, 8.6%)
Kolenda et al., 2020 (41)	15	99	Critically ill	Mainly amoxicillin and clavulanic acid or third generation cephalosporins associated with macrolides
Li et al., 2020 (43)	148	225	Not specific: General	Moxifloxacin and others
Liu et al., 2020 (45)	128	140	Not specific: General	NR
Liu et al., 2021 (47)	792	1123	Not specific: General	Fluoroquinolones (59.3%) Moxifloxacin (36.4%)
Mousav Movahed et al., 2021 (51)	243	854	Not specific: General including critically ill	NR
Nassir et al., 2021 (52)	82	100	Not specific: General including critically ill	NR
Nebreda et al., 2020 (53)	84	712	Not specific: General including critically ill	NR
Pulia et al., 2021 (54)	27	73	Not specific: General	NR
Quatuccio et al., 2020 (55)	9	69	SOC overall	NR
Rothe et al., 2020 (58)*	22	56	critically ill	Various mentioned: most common piperacillin-tazobactam
Rothe et al., 2020 (58)*	109	135	Not specific: General including critically ill	Various mentioned: most common ampicillin/sulbactam
Seaton et al., 2020 (59)*	219	421	Not specific: General including critically ill	Various antibiotics, most common including: doxycycline, amoxicillin, co-amoxiclav, piperacillin-tazobactam and vancomycin among others
Seaton et al., 2020 (59)*	71	110	Critically ill	Various antibiotics, most common including: meropenem, piperacillin-tazobactam and co-amoxiclav
Shah et al., 2020 (60)	17	26	Not specific: General including critically ill	Majority received vancomycin, tazocin, cefepime, or ceftriaxone
Shao et al., 2020 (61)	81	126	Not specific: General l	NR
Sharifipour et al., 2020 (62)	19	19	Critically ill	NR
Soogard et al., 2021 (65)	71	162	Not specific: General including critically ill	Antibiotics or antifungals
Soogard et al., 2021 (65)	36	41	Critically ill	Antibiotics or antifungals
Staub et al., 2021 (66)	86	131	Not specific: General	NR
Stevens et al., 2021 (67)	33	346	Not specific: General including critically ill	Most common: piperacillin-tazobactam, ceftriaxone and azithromycin
Tang et al., 2021 (68)	58	72	Not specific: General including critically ill	Levofloxacin = 21, moxifloxacin = 22, levofloxacin swapped to moxifloxacin = 5, among others
Towsend et al., 2020 (70)*	84	117	Not specific: General including critically ill	Treated as lower respiratory tract infection
Vanhomwegen et al., 2021 (71)*	54	66	Critically ill	NR
Vaughn et al., 2021 (72)	965	1705	Not specific: General	The most commonly prescribed empirical antibiotics were ceftriaxone (663/1705, 38.9%), vancomycin (235/1705, 13.8%), doxycycline (185/1705, 10.9%), and cefepime (177/1705, 10.4%)
Wan et al., 2020 (73)	59	135	Not specific: General including critically ill	NR
Wan et al., 2020 (73)	35	40	Severe and critically ill	NR
Wang et al., 2020 (74)	27	28	Not specific: General including critically ill	NR
Wang et al., 2020 (74)	14	14	Critically ill	NR
Yang et al., 2020 (78)	172	251	Not specific: General	NR
Zhang et al., 2020 (82)	131	134	Critically ill	NR
Zhang et al., 2021 (83)	21	91	Elderly	NR
Zhang et al., 2020 (84)	251	365	Not specific: General	NR
Zhao et al., 2020 (85)	783	1000	Not specific: General including critically ill	NR
Xu et al., 2021 (77)	58	62	Critically ill	NR

n: number of patients prescribed antibiotics. N: total number of patients. ITU: intensive treatment unit

**Clearly referred to as empirical use

Full list of references are in Additional file 1: Material S4

**Fig. 5:**
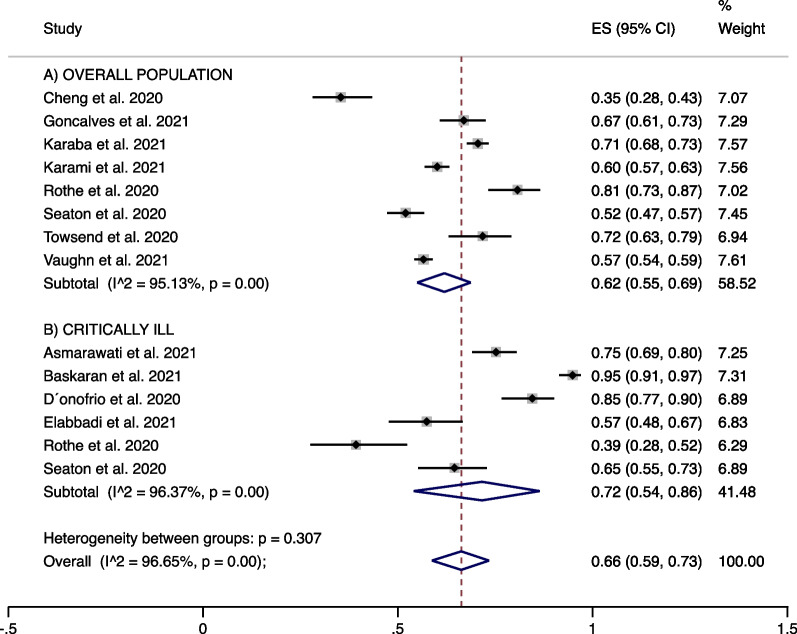
Studies clearly describing empirical antibiotic use

Eleven studies (12.9%) clearly described empirical antibiotic use. A sub-analysis of these papers found that overall empirical antibiotic use was 62% (eight studies, 95% CI 55 to 69%; I^2^ 95.1%) (Fig. 5A) and in critically ill patients was 66% (six studies, 95% CI 58 to 73%; I^2^ 96.6%) (Fig. 5B).

### Antibiotic stewardship

Eleven studies specifically stated that empirical antibiotics were commenced of which five described decision-making processes regarding antibiotics.

Cheng et al. [9] mentioned that 52/147 (35%) patients received empirical antibiotics and that 19 (37%) received antibiotics for more than a week despite negative cultures. The median length of course of empirical antibiotics was seven (IQR = 5 to 12) days.

Rothe et al. [10] described the implementation of an antibiotic stewardship standard operational procedure in their institution in which initiation of antibiotic therapy was recommended only in cases of clinically suspected infection (narrow spectrum aminopenicillin/beta-lactamase inhibitor combination). However, decisions regarding stewardship were at the clinician’s discretion. The most used antibiotic scheme during the observation period were ampicillin/sulbactam (41.5%) and piperacillin/ tazobactam (19.3%) with or without azithromycin. Median duration of were variable being longer in the case of piperacillin/ tazobactam 10 (range 3 to 26) days. Interestingly, azithromycin was not included in the guidelines, although it was used in 43 patients (31.9%) as combination therapy.

Townsend et al. [11] described 84 patients treated empirically for respiratory bacterial co-infection of which 78 (92.9%) received monotherapy. All treatment was initially intravenous, and an oral switch took place in only 34 (40.5%) cases. The median durations of intravenous and oral therapies were five days (range 1 to 14) and three days (range 1 to 4) respectively.

Karami et al. [12] described the adherence to local guidelines on empiric antibiotic therapy in their institution. Mean adherence was 60.3% (range 45.3% to 74.7%) on the first day of admission showing that 556 of 925 (60.1%) patients were prescribed empirical antibiotics. However, the rate of antibiotic prescribing increased after seven days of admission to 669 (72.3%). Confirmed bacterial co-infection was confirmed only in 12/925 (1.2%) patients. Regarding length of antibiotics use, 467 of 555 (84.1%) had five days of antibiotics. Intravenous antibiotics exceeded 48 h in 413 patients who started antibiotic treatment on the first day of admission and oral switched were performed in 9.9% of those.

Vaugh et al. [13] described that of the patients who received empiric antibiotic therapy (N = 965), the majority (612, 63.4%) received antibiotics targeting community-acquired microorganism. The median of duration of inpatient antibiotic was three days (IQR, 2 to 6 days) in the patients receiving antibiotics. Total days of inpatient antibiotic therapy was 4158 days/1000 patients.

The remainder of these studies (Seaton et al. [14], Baskaraban et al. [15], Goncalves et al. [16], Karaba et al. [17], Asmarawati et al. [18], D’onofrio et al. [19] and Elabbadi [20]) did not describe empirical antibiotic duration or any specific criteria for stopping treatment, although they do state that local guidelines for empirical antibiotic use in COVID-19 pneumonitis should be applied.

## Discussion

In the absence of clear guidance on when to give empirical antibiotic therapy to patients admitted to hospital with COVID-19 pneumonia, clinicians face a dilemma. In the context of a global pandemic and given the potential risks of antibiotic treatment to patients and to public health, it is essential that the best available evidence is used to support clinicians on the front line to appropriately balance risks to patients and to the wider public.

We analysed bacterial co-infection in different ways in order to evaluate how estimates may vary depending on authors’ definitions. Based only on author descriptions, we found a prevalence of bacterial co-infection of 12% (95% CI 8 to 16%) in the overall population. Interestingly, we observed that bacterial co-infection was lower when including only studies with clear definitions of bacterial co-infection (overall population 4% (95% CI 3 to 6%), critically ill patients 12% (95% CI 4 to 22%)). Our results are similar to that found by other authors who have evaluated co-infections in patients with COVID-19. For example, Rawson et al. [21] conducted a meta-analysis which found a prevalence of bacterial and fungal coinfection of 8%. Langford et al. evaluated bacterial co-infection at presentation and after presentation of COVID-19, finding a prevalence of 3.5% (95% CI 0.4 to 6.7%) for primary co-infection and 14.3% (95% CI 9.6 to 18.9%) for secondary (nosocomial) co-infection [22].

It is important to acknowledge that our estimates of the prevalence of bacterial co-infection prevalence were derived from a number of different definitions, as provided by the authors of the source papers. This is relevant, as although microbiological cultures are the gold standard for diagnosis, these are neither quick nor universally available tools on which to base prescribing decisions, particularly in patients with severe disease.

Our study also finds that, as expected, the overall use of antibiotics in patients with COVID-19 is high compared to the estimated prevalence of bacterial co-infection. We identified a prevalence of empirical antibiotic use of 62% (95%CI 55 to 29%). These estimates are similar to those of Langford et al. [22], who found an overall prevalence of antibiotic use of 71.9% (95% CI 56.1 to 87.7%). Our slightly lower estimates may be explained by having retrieved studies nearly one and a half years after the start of the pandemic. This could reflect changes in empirical practice through increased experience in managing COVID-19, coupled with more data being available to inform evidence-based practice regarding antibiotic use. Furthermore, the previous study provided estimates of antibiotic use based only on patients with culture confirmed bacterial co-infections, while we included all COVID-19 patients that were considered to have an infection in our estimate, regardless of whether bacterial co-infections were ultimately confirmed. In doing so, we have sought to reflect real world practice, and we suggest that estimates of overall empirical antibiotic use that are not restricted to patients with confirmed infections are important to understanding the need for, and potential impact of, antimicrobial stewardship tools and strategies as part of the response to the COVID-19 pandemic.

The final aim of our study was to identify to what degree decisions to stop empirically prescribed antibiotics were being made according to any defined criteria. This aspect has not been addressed previously in published systematic reviews and meta-analyses. Despite terms related to stewardship being specifically included in our search strategy and despite meticulously reading all included citations in full, including discussion sections, we found very little information on stewardship measures. We found this absence of information particularly notable given that antimicrobial resistance is widely acknowledged as being one of the most serious public health challenges of our times [23–25]. Whilst we acknowledge that case reports and series are generally more concerned with describing the clinical and demographic characteristics of their patients, it is nevertheless disappointing that the large observed differences between confirmed bacterial co-infection and frequency of antibiotic use does not prompt authors to consider this matter more prominently in their discussions. Despite these deficits in the current literature, we assert that it is of fundamental importance to preserve any goals and achievements relating to antibiotic stewardship established prior to the COVID-19 pandemic. Several antibiotic stewardship programs such as ARK (Antibiotic Review Kit) and TARGET (Treat Antibiotics Responsibly, Guidance, Education, Tools) have been shown to be both feasible and acceptable in supporting the safe discontinuation of antibiotics post-prescription in acute hospital settings [26, 27]. These are just two examples of efforts that must be continued, particularly in the current climate of highly prevalent empirical use of antibiotics during a viral pandemic in which the prevalence of confirmed bacterial co-infection appears to be low.

Our study has some important limitations. The most important being that the COVID-19 pandemic has given rise to an unprecedented situation in the scientific world in terms of a seemingly exponential increase in the volume of related publications over a very short time. Thus, at the time of writing there are likely to be additional studies that would have qualified for inclusion. This rapidity of publication would necessitate updating searches and analysis on as much as a weekly basis, which we suggest would be unrealistic for a piece of peer-reviewed work such as this. It is reassuring to know, however, that other groups pursuing similar research questions [21, 22, 28] have found similar results despite not having included the same studies or conducting searches that cover the same dates. To the best of our knowledge, the present systematic review is the currently most up to date systematic review of this subject, presenting data from more than 30,000 patients from studies identified through an exhaustive search strategy. Another important point to highlight is that most of the included studies in this systematic review are from high income countries, and caution should therefore be exercised when generalising from our results to other settings. Further studies should analyse how COVID-19 has affected antibiotic use in low- and middle-income countries, where the burden of drug-resistant infections is greatest [24].

Another important limitation is one that is inherent to this type of analysis. There is a consensus that the methodology for systematic reviews of prevalence data is not well developed, with a notable lack of methodological and reporting guidance for systematic reviews of prevalence data [29, 30]. Thus, in most cases authors present adapted or de novo tools to assess the quality of the prevalence data that will be included in the analysis, regardless of the study design [22, 28, 31]. In our case, we used a tool that has been developed acknowledging that prevalence data can come from different study designs, however we cannot make an overall assessment of risk of bias [8]. Prevalence metanalysis have also the risk of presenting high level of heterogeneity. We have sought to address the high level of heterogeneity by using statistical correction as well as performing subgroup analyses. Nevertheless, caution should be exercised with extrapolation to specific contexts.

## Conclusion

In this study we have reported bacterial co-infection and antibiotic use during the first 18 months of the SARS-CoV-2 pandemic. This work can help clinicians to reflect on and understand the initial response to a global pandemic of a novel respiratory virus. Our results show that there is currently insufficient evidence to support the use of empirical use of antibiotics in most hospitalised patients with COVID-19, as the overall proportion of bacterial co-infection in these patients is low. Furthermore, as the use of antibiotics in COVID-19 appears to have been largely empirical, it is necessary to identify clinical and laboratory markers and to formulate guidelines to promote more targeted administration of antibiotics in patients admitted to hospital with COVID-19.

## Supplementary Information

**Additional file 1. Material S1.** PRISMA check list. **Material S2.** Search Strategy. **Material S3.** Quality Assessment of included studies. **Material S4.** Reference list of included studies.

## Data Availability

All data generated or analysed during this study are included in this published article [and its supplementary information files].
