# Supplementary material for: Bacterial co-infection and antibiotic stewardship in patients with COVID-19: a systematic review and meta-analysis
Source: BMC Infect Dis. 2023 Jan 9;23:14. doi: 10.1186/s12879-022-07942-x (PMC9828368; doi:10.1186/s12879-022-07942-x)
Supplement: Supplementary file 1 — Additional file 1. Material S1. PRISMA check list. Material S2. Search Strategy. Material S3. Quality Assessment of included studies. Material S4. Reference list of included studies. [file 12879_2022_7942_MOESM1_ESM.docx]

**MATERIAL S1**: PRISMA CHECK LIST

| **Section/topic** | **#** | **Checklist item** | **Reported on page #** |
| --- | --- | --- | --- |
| **TITLE** | | |  |
| Title | **1** | Identify the report as a systematic review, meta-analysis, or both. | 1 |
| **ABSTRACT** | | |  |
| Structured summary | **2** | Provide a structured summary including, as applicable: background; objectives; data sources; study eligibility criteria, participants, and interventions; study appraisal and synthesis methods; results; limitations; conclusions and implications of key findings; systematic review registration number. | 2 |
| **INTRODUCTION** | | |  |
| Rationale | **3** | Describe the rationale for the review in the context of what is already known. | 2-4 |
| Objectives | **4** | Provide an explicit statement of questions being addressed with reference to participants, interventions, comparisons, outcomes, and study design (PICOS). | 2-4 |
| **METHODS** | | |  |
| Protocol and registration | **5** | Indicate if a review protocol exists, if and where it can be accessed (e.g., Web address), and, if available, provide registration information including registration number. | 3 |
| Eligibility criteria | **6** | Specify study characteristics (e.g., PICOS, length of follow-up) and report characteristics (e.g., years considered, language, publication status) used as criteria for eligibility, giving rationale. | 3-4 |
| Information sources | **7** | Describe all information sources (e.g., databases with dates of coverage, contact with study authors to identify additional studies) in the search and date last searched. | 3-4 |
| Search | **8** | Present full electronic search strategy for at least one database, including any limits used, such that it could be repeated. | 3 and appendix |
| Study selection | **9** | State the process for selecting studies (i.e., screening, eligibility, included in systematic review, and, if applicable, included in the meta-analysis). | 3-4 |
| Data collection process | **10** | Describe method of data extraction from reports (e.g., piloted forms, independently, in duplicate) and any processes for obtaining and confirming data from investigators. | 4-5 |
| Data items | **11** | List and define all variables for which data were sought (e.g., PICOS, funding sources) and any assumptions and simplifications made. | 4-5 |
| Risk of bias in individual studies | **12** | Describe methods used for assessing risk of bias of individual studies (including specification of whether this was done at the study or outcome level), and how this information is to be used in any data synthesis. | 4-5 |
| Summary measures | **13** | State the principal summary measures (e.g., risk ratio, difference in means). | 4-5 |
| Synthesis of results | **14** | Describe the methods of handling data and combining results of studies, if done, including measures of consistency (e.g., I^2^) for each meta-analysis. | 4-5 |
| Risk of bias across studies | **15** | Specify any assessment of risk of bias that may affect the cumulative evidence (e.g., publication bias, selective reporting within studies). | 5 |
| Additional analyses | **16** | Describe methods of additional analyses (e.g., sensitivity or subgroup analyses, meta-regression), if done, indicating which were pre-specified. | 4-5 |
| **RESULTS** | | |  |
| Study selection | **17** | Give numbers of studies screened, assessed for eligibility, and included in the review, with reasons for exclusions at each stage, ideally with a flow diagram. | 5-6  Figure 1 |
| Study characteristics | **18** | For each study, present characteristics for which data were extracted (e.g., study size, PICOS, follow-up period) and provide the citations. | Table 1 |
| Risk of bias within studies | **19** | Present data on risk of bias of each study and, if available, any outcome level assessment (see item 12). | Appendix |
| Results of individual studies | **20** | For all outcomes considered (benefits or harms), present, for each study: (a) simple summary data for each intervention group (b) effect estimates and confidence intervals, ideally with a forest plot. | Figure 3,4,5 |
| Synthesis of results | **21** | Present results of each meta-analysis done, including confidence intervals and measures of consistency. | Figure 3,4,5 |
| Risk of bias across studies | **22** | Present results of any assessment of risk of bias across studies (see Item 15). | NA |
| Additional analysis | **23** | Give results of additional analyses, if done (e.g., sensitivity or subgroup analyses, meta-regression [see Item 16]). | Figure 5 |
| **DISCUSSION** | | |  |
| Summary of evidence | **24** | Summarize the main findings including the strength of evidence for each main outcome; consider their relevance to key groups (e.g., healthcare providers, users, and policy makers). | 8-11 |
| Limitations | **25** | Discuss limitations at study and outcome level (e.g., risk of bias), and at review-level (e.g., incomplete retrieval of identified research, reporting bias). | 8-11 |
| Conclusions | **26** | Provide a general interpretation of the results in the context of other evidence, and implications for future research. | 8-11 |
| **FUNDING** | | |  |
| Funding | **27** | Describe sources of funding for the systematic review and other support (e.g., supply of data); role of funders for the systematic review. | 11 and journal submission |

**MATERIAL S2**: SEARCH STRATEGY

Pubmed 3/5/2021

| Search | Query | Results |
| --- | --- | --- |
| #32 | **#20 AND #31** | [2,335](https://pubmed.ncbi.nlm.nih.gov/?term=%2320+AND+%2331&ac=no&sort=relevance) |
| #31 | **#21 OR #22 OR #23 OR #24 OR #25 OR #26 OR #27 OR #28 OR #29 OR #30** | [105,534](https://pubmed.ncbi.nlm.nih.gov/?term=%2321+OR+%2322+OR+%2323+OR+%2324+OR+%2325+OR+%2326+OR+%2327+OR+%2328+OR+%2329+OR+%2330&ac=no&sort=relevance) |
| #30 | **Antibiotic Steward*[tiab]** | [2,542](https://pubmed.ncbi.nlm.nih.gov/?term=Antibiotic+Steward%2A%5Btiab%5D&ac=no&sort=relevance) |
| #29 | **Antimicrobial Stewardship[Mesh]** | [2,030](https://pubmed.ncbi.nlm.nih.gov/?term=Antimicrobial+Stewardship%5BMesh%5D&ac=no&sort=relevance) |
| #28 | **Superinfect*[tiab]** | [6,740](https://pubmed.ncbi.nlm.nih.gov/?term=Superinfect%2A%5Btiab%5D&ac=no&sort=relevance) |
| #27 | **Bacterial Infection*[tiab]** | [44,335](https://pubmed.ncbi.nlm.nih.gov/?term=Bacterial+Infection%2A%5Btiab%5D&ac=no&sort=relevance) |
| #26 | **Secondary Infection*[tiab]** | [5,644](https://pubmed.ncbi.nlm.nih.gov/?term=Secondary+Infection%2A%5Btiab%5D&ac=no&sort=relevance) |
| #25 | **Mixed Infection*[tiab]** | [7,623](https://pubmed.ncbi.nlm.nih.gov/?term=Mixed+Infection%2A%5Btiab%5D&ac=no&sort=relevance) |
| #24 | **Cross Infect*[tiab]** | [3,723](https://pubmed.ncbi.nlm.nih.gov/?term=Cross+Infect%2A%5Btiab%5D&ac=no&sort=relevance) |
| #23 | **Coinfect*[tiab]** | [32,486](https://pubmed.ncbi.nlm.nih.gov/?term=Coinfect%2A%5Btiab%5D&ac=no&sort=relevance) |
| #22 | **Co-Infect*[tiab]** | [18,241](https://pubmed.ncbi.nlm.nih.gov/?term=Co-Infect%2A%5Btiab%5D&ac=no&sort=relevance) |
| #21 | **Coinfection[Mesh]** | [12,079](https://pubmed.ncbi.nlm.nih.gov/?term=Coinfection%5BMesh%5D&ac=no&sort=relevance) |
| #20 | **#1 OR #2 OR #3 OR #4 OR #5 OR #6 OR #7 OR #8 OR #9 OR #10 OR #11 OR #12 OR #13 OR #14 OR #15 OR #16 OR #17 OR #18 OR #19** | [145,005](https://pubmed.ncbi.nlm.nih.gov/?term=%231+OR+%232+OR+%233+OR+%234+OR+%235+OR+%236+OR+%237+OR+%238+OR+%239+OR+%2310+OR+%2311+OR+%2312+OR+%2313+OR+%2314+OR+%2315+OR+%2316+OR+%2317+OR+%2318+OR+%2319&ac=no&sort=relevance) |
| #19 | **N-Cov[tiab]** | [18](https://pubmed.ncbi.nlm.nih.gov/?term=N-Cov%5Btiab%5D&ac=no&sort=relevance) |
| #18 | **CV-19*[tiab]** | [72](https://pubmed.ncbi.nlm.nih.gov/?term=CV-19%2A%5Btiab%5D&ac=no&sort=relevance) |
| #17 | **CV19*[tiab]** | [20](https://pubmed.ncbi.nlm.nih.gov/?term=CV19%2A%5Btiab%5D&ac=no&sort=relevance) |
| #16 | **HCov*[tiab]** | [1,011](https://pubmed.ncbi.nlm.nih.gov/?term=HCov%2A%5Btiab%5D&ac=no&sort=relevance) |
| #15 | **Corono Virus[tiab]** | [1](https://pubmed.ncbi.nlm.nih.gov/?term=Corono+Virus%5Btiab%5D&ac=no&sort=relevance) |
| #14 | **Virus Corona[tiab]** | [2,520](https://pubmed.ncbi.nlm.nih.gov/?term=Virus+Corona%5Btiab%5D&ac=no&sort=relevance) |
| #13 | **Coronovir*[tiab]** | [53](https://pubmed.ncbi.nlm.nih.gov/?term=Coronovir%2A%5Btiab%5D&ac=no&sort=relevance) |
| #12 | **Coronavir*[tiab]** | [62,664](https://pubmed.ncbi.nlm.nih.gov/?term=Coronavir%2A%5Btiab%5D&ac=no&sort=relevance) |
| #11 | **(Pneumonia[tiab] AND Wuhan[tiab] AND 2019[tiab])** | [1,097](https://pubmed.ncbi.nlm.nih.gov/?term=%28Pneumonia%5Btiab%5D+AND+Wuhan%5Btiab%5D+AND+2019%5Btiab%5D%29&ac=no&sort=relevance) |
| #10 | **SARSCoV2[tiab]** | [35,412](https://pubmed.ncbi.nlm.nih.gov/?term=SARSCoV2%5Btiab%5D&ac=no&sort=relevance) |
| #9 | **SARS-CoV2[tiab]** | [1,816](https://pubmed.ncbi.nlm.nih.gov/?term=SARS-CoV2%5Btiab%5D&ac=no&sort=relevance) |
| #8 | **SARS-CoV-2[tiab]** | [41,303](https://pubmed.ncbi.nlm.nih.gov/?term=SARS-CoV-2%5Btiab%5D&ac=no&sort=relevance) |
| #7 | **2019-nCoV[tiab]** | [1,609](https://pubmed.ncbi.nlm.nih.gov/?term=2019-nCoV%5Btiab%5D&ac=no&sort=relevance) |
| #6 | **COVID19*[tiab]** | [108,423](https://pubmed.ncbi.nlm.nih.gov/?term=COVID19%2A%5Btiab%5D&ac=no&sort=relevance) |
| #5 | **COVID-19[tiab]** | [112,939](https://pubmed.ncbi.nlm.nih.gov/?term=COVID-19%5Btiab%5D&ac=no&sort=relevance) |
| #4 | **Corona Virus[tiab]** | [1,926](https://pubmed.ncbi.nlm.nih.gov/?term=Corona+Virus%5Btiab%5D&ac=no&sort=relevance) |
| #3 | **COVID-19[Mesh]** | [74,161](https://pubmed.ncbi.nlm.nih.gov/?term=COVID-19%5BMesh%5D&ac=no&sort=relevance) |
| #2 | **SARS-CoV-2[Mesh]** | [57,191](https://pubmed.ncbi.nlm.nih.gov/?term=SARS-CoV-2%5BMesh%5D&ac=no&sort=relevance) |
| #1 | **Coronavirus[Mesh]** | [69,584](https://pubmed.ncbi.nlm.nih.gov/?term=Coronavirus%5BMesh%5D&ac=no&sort=relevance) |

EMBase 3/5/2021

Embase <1974 to 2021 April 30>

1 exp Coronavirinae/ 47151

2 exp SARS Coronavirus/ 6527

3 exp Coronavirus Infection/ 126181

4 COVID-19.ti,ab. 105986

5 (Coron* adj1 Virus).ti,ab. 2109

6 COVID19*.ti,ab. 1606

7 2019-nCoV.ti,ab. 1234

8 SARS-CoV-2.ti,ab. 33630

9 SARS-CoV2.ti,ab. 1680

10 SARSCoV2.ti,ab. 74

11 (Pneumonia and Wuhan and "2019").ti,ab. 1086

12 Coronavir*.ti,ab. 56050

13 Coronovir*.ti,ab. 57

14 HCov*.ti,ab. 1046

15 CV19*.ti,ab. 47

16 CV-19*.ti,ab. 189

17 N-Cov.ti,ab. 29

18 or/1-17 156336

19 exp Mixed Infection/ 39893

20 Co-Infect*.ti,ab. 27349

21 Coinfect*.ti,ab. 21503

22 (Cross* adj1 Infect*).ti,ab. 3452

23 (Mix* adj1 Infection*).ti,ab. 8872

24 Secondary Infection*.ti,ab. 7387

25 Bacterial Infection*.ti,ab. 58489

26 Superinfect*.ti,ab. 7527

27 exp Antimicrobial Stewardship/ 6096

28 Antibiotic Steward*.ti,ab. 3494

29 Antimicrobial Steward*.ti,ab. 6217

30 or/19-29 149650

31 18 and 30 3135

CINAHL (EBSCO) 3/5/2021

| **#** | **Query** | **Results** |
| --- | --- | --- |
| S32 | S19 AND S31 | 334 |
| S31 | S20 OR S21 OR S22 OR S23 OR S24 OR S25 OR S26 OR S27 OR S28 OR S29 OR S30 | 14,195 |
| S30 | TI (Antibacterial* N1 Steward*) OR AB (Antibacterial* N1 Steward*) | 4 |
| S29 | TI (Antibiotic* N1 Steward*) OR AB (Antibiotic* N1 Steward*) | 1,001 |
| S28 | (MH "Antimicrobial Stewardship") | 935 |
| S27 | TI Superinfect* OR AB Superinfect* | 379 |
| S26 | TI (Bacterial N1 Infection*) OR AB (Bacterial N1 Infection*) | 5,294 |
| S25 | TI (Secondary N1 Infection*) OR AB (Secondary N1 Infection*) | 1,364 |
| S24 | TI (Mix* N1 Infect*) OR AB (Mix* N1 Infect*) | 483 |
| S23 | TI (Cross* N1 Infect*) OR AB (Cross* N1 Infect*) | 791 |
| S22 | TI Coinfect* OR AB Coinfect* | 2,261 |
| S21 | TI Co-Infect* OR AB Co-Infect* | 2,217 |
| S20 | (MH "Coinfection") | 1,825 |
| S19 | S1 OR S2 OR S3 OR S4 OR S5 OR S6 OR S7 OR S8 OR S9 OR S10 OR S11 OR S12 OR S13 OR S14 OR S15 OR S16 OR S17 OR S18 | 29,885 |
| S18 | TI N-Cov* OR AB N-Cov* | 3 |
| S17 | TI CV-19* OR AB CV-19* | 52 |
| S16 | TI CV-19* OR AB CV-19* | 0 |
| S15 | TI CV19* OR AB CV19* | 1 |
| S14 | TI HCov* OR AB HCov* | 74 |
| S13 | TI Coronovir* OR AB Coronovir* | 8 |
| S12 | TI Coronavir* OR AB Coronavir* | 12,028 |
| S11 | TI (Pneumonia AND Wuhan AND 2019) OR AB (Pneumonia AND Wuhan AND 2019) | 205 |
| S10 | TI SARSCoV2 OR AB SARSCoV2 | 7 |
| S9 | TI SARS-CoV2 OR AB SARS-CoV2 | 147 |
| S8 | TI SARS-CoV-2 OR AB SARS-CoV-2 | 4,633 |
| S7 | TI 2019-nCoV OR AB 2019-nCoV | 193 |
| S6 | TI COVID19* OR AB COVID19* | 172 |
| S5 | TI COVID-19* OR AB COVID-19* | 24,878 |
| S4 | TI (Coron* N1 Virus) OR AB (Coron* N1 Virus) | 370 |
| S3 | (MH "COVID-19") | 9,764 |
| S2 | (MH "Coronavirus Infections+") | 16,731 |
| S1 | (MH "Coronavirus+") | 1,093 |

Cochrane Library (Wiley) 4/5/2021

ID Search Hits

#1 MeSH descriptor: [Coronavirus] explode all trees 264

#2 MeSH descriptor: [SARS-CoV-2] explode all trees 251

#3 MeSH descriptor: [COVID-19] explode all trees 337

#4 (Coron* NEAR/1 Virus):ti,ab,kw 214

#5 COVID-19:ti,ab,kw 4915

#6 COVID19:ti,ab,kw 304

#7 "2019-nCoV":ti,ab,kw 10

#8 SARS-CoV-2:ti,ab,kw 222

#9 SARS-CoV2:ti,ab,kw 54

#10 SARSCoV2:ti,ab,kw 185

#11 (Pneumonia AND Wuhan AND 2019):ti,ab,kw 93

#12 Coronavir*:ti,ab,kw 2906

#13 Coronovir*:ti,ab,kw 1

#14 HCov*:ti,ab,kw 16

#15 CV19*:ti,ab,kw 0

#16 CV-19*:ti,ab,kw 12

#17 N-Cov:ti,ab,kw 16

#18 #1 OR #2 OR #3 OR #4 OR #5 OR #6 OR #7 OR #8 OR #9 OR #10 OR #11 OR #12 OR #13 OR #14 OR #15 OR #16 OR #17 5273

#19 MeSH descriptor: [Coinfection] explode all trees 178

#20 Co-Infect*:ti,ab,kw 953

#21 Coinfect*:ti,ab,kw 1587

#22 (Cross* NEAR/1 Infect*):ti,ab,kw 1337

#23 (Mix* NEAR/1 Infection*):ti,ab,kw 651

#24 (Secondary NEAR/3 Infection*):ti,ab,kw 1446

#25 (Bacterial NEAR/3 Infection*):ti,ab,kw 7579

#26 Superinfect*:ti,ab,kw 548

#27 MeSH descriptor: [Antimicrobial Stewardship] explode all trees 30

#28 (Antibiotic NEAR/1 Steward*):ti,ab,kw 128

#29 (Antibacterial NEAR/1 Steward*):ti,ab,kw 3

#30 #19 OR #20 OR #21 OR #22 OR #23 OR #24 OR #25 OR #26 OR #27 OR #28 OR #29 12206

#31 #18 AND #30 166

LILACS (BVS Eng) 4/5/2021

| [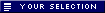](javascript:void(AnySelected())) |  |  |  | [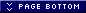](http://bases.bireme.br/cgi-bin/wxislind.exe/iah/online/#bottom) |
| --- | --- | --- | --- | --- |

| \| Database : \| **LILACS** \| \| --- \| --- \| \| Search on : \| **(MH Coronavirus OR SARS-CoV-2 OR SARS-CoV2 OR Coronavir$ OR MH Coronavirus Infections OR MH Betacoronavirus OR Corona OR Covid-19 OR Covid19 OR 2019-nCoV$ OR ((Pneumonia OR Neumonia) AND (Wuhan AND 2019))) AND (MH Coinfection OR Coinfect$ OR Coinfecção OR Coinfección OR Co-Infec$ OR Bacterial OR MH Antimicrobial Stewardship OR Stewardship OR Superinfec$ OR Antimicrobiano$) [Words]** \| \| References found : \| **101** [[refine](http://bases.bireme.br/cgi-bin/wxislind.exe/iah/online/#refine)] \| |
| --- | --- | --- | --- | --- | --- | --- |

Global Health 4/5/2021

Global Health <1910 to 2021 Week 17>

1 Coronavirus.mp. 33074

2 SARS-CoV-2.mp. 10834

3 COVID-19.mp. 25171

4 (Coron* adj1 Virus).mp. 682

5 COVID19.mp. 275

6 2019-nCoV.mp. 915

7 SARS-CoV2.mp. 401

8 SARSCoV2.mp. 9

9 (Pneumonia and Wuhan and "2019").ti,ab. 412

10 (Coron* and "2019").ti,ab. 9325

11 or/1-10 33910

12 Co-Infect*.ti,ab. 11538

13 Coinfect*.ti,ab. 9179

14 (Cross* adj1 Infect*).ti,ab. 1986

15 (Mix* adj1 Infect*).ti,ab. 6888

16 (Secondary adj1 Infect*).ti,ab. 3547

17 (Bacterial adj1 Infect*).ti,ab. 14522

18 Superinfect*.ti,ab. 2418

19 (Antibiotic adj2 Steward*).ti,ab. 1202

20 (Antibacterial adj2 Steward*).ti,ab. 11

21 (Antimicrobial adj2 Steward*).ti,ab. 2362

22 or/12-21 51171

23 11 and 22 827

**MATERIAL S3**: QUALITY ASSESSMENT

**Table**: The Joanna Briggs Institute (JBI) Critical Appraisal Checklist for descriptive cross-sectional/prevalence data.

| Author | Was the sample frame appropriate to address the target population? | Were study participants sampled in an appropriate way? | Was the sample size adequate? | Were the study subjects and the setting described in detail? | Was the data analysis conducted with sufficient coverage of the identified sample? | Were valid methods used for the identification of the condition? | Was the condition measured in a standard, reliable way for all participants? | Was there appropriate statistical analysis? | Was the response rate adequate, and if not, was the low response rate managed appropriately? |
| --- | --- | --- | --- | --- | --- | --- | --- | --- | --- |
| Alharty et al. 2020 (1) | Yes | Yes | N/A | Yes | Yes | Yes | Yes | NA | NA |
| Allou et al. 2021(2) | Yes | Yes | N/A | Yes | Yes | Yes | Yes | NA | NA |
| Amit et al. 2020 (3) | Yes | Yes | Yes | Yes | Yes | Yes | Yes | Yes | Yes |
| Asmarawati et al. 2021(4) | Yes | Yes | N/A | Yes | Yes | Yes | Yes | NA | NA |
| Ayding Bahat et al. 2020(5) | Yes | Yes | N/A | Yes | Yes | Yes | Yes | NA | NA |
| Balena et al. 2020(6) | Yes | Yes | N/A | Yes | Yes | Yes | Yes | NA | NA |
| Baraboutis et al. 2020(7) | Yes | Yes | N/A | Yes | Yes | Yes | Yes | NA | NA |
| Bardi et al. 2021(8) | Yes | Yes | N/A | Yes | Yes | Yes | Yes | NA | NA |
| Barrasa et al. 2020 (9) | Yes | Yes | N/A | Yes | Yes | Yes | Yes | NA | NA |
| Barry et al. 2020(10) | Yes | Yes | N/A | Yes | Yes | Yes | Yes | NA | NA |
| Basakaran et al. 2021 (11) | Yes | Yes | Yes | Yes | Yes | Yes | Yes | NA | NA |
| Bhatt et al. 2021(12) | Yes | Yes | Yes | Yes | Yes | Yes | Yes | NA | NA |
| Buckner et al. 2020(13) | Yes | Yes | Yes | Yes | Yes | Yes | Yes | NA | NA |
| Chen et al. 2020(14) | Yes | Yes | N/A | Yes | Yes | Yes | Yes | NA | NA |
| Chen et al. 2021(15) | Yes | Yes | N/A | Yes | Yes | Yes | Yes | NA | NA |
| Cheng et al. 2020(16) | Yes | Yes | N/A | Yes | Yes | Yes | Yes | NA | NA |
| Chengy et al. 2020(17) | Yes | Yes | N/A | Yes | Yes | Yes | Yes | NA | NA |
| Chong et al. 2021(18) | Yes | Yes | N/A | Yes | Yes | Yes | Yes | NA | NA |
| Choubey at al. 2021(19) | Yes | Yes | N/A | Yes | Yes | Yes | Yes | NA | NA |
| Contou et al. 2020 (20) | Yes | Yes | N/A | Yes | Yes | Yes | Yes | NA | NA |
| D’onofrio et al 2020(21) | Yes | Yes | N/A | Yes | Yes | Yes | Yes | NA | NA |
| Desai et al. 2020(22) | Yes | Yes | N/A | Yes | Yes | Yes | Yes | NA | NA |
| Dolci et al. 2020(23) | Yes | Yes | N/A | Yes | Yes | Yes | Yes | NA | NA |
| Ekadashi et al. 2021(24) | Yes | Yes | N/A | Yes | Yes | Yes | Yes | NA | NA |
| Elabbadi et al. 2021(25) | Yes | Yes | N/A | Yes | Yes | Yes | Yes | NA | NA |
| Falcone et al. 20201(26) | Yes | Yes | N/A | Yes | Yes | Yes | Yes | NA | NA |
| Fan et al. 2021(27) | Yes | Yes | N/A | Yes | Yes | Yes | Yes | NA | NA |
| Garcia-Vidal et al. 2021(28) | Yes | Yes | N/A | Yes | Yes | Yes | Yes | NA | NA |
| Gayam et al. 2020 (29) | Yes | Yes | N/A | Yes | Yes | Yes | Yes | NA | NA |
| Goncalves et al. 2021 (30) | Yes | Yes | N/A | Yes | Yes | Yes | Yes | NA | NA |
| Guan et al. 2020 (31) | Yes | Yes | N/A | Yes | Yes | Yes | Yes | NA | NA |
| He et al 2020 .(32) | Yes | Yes | N/A | Yes | Yes | Yes | Yes | NA | NA |
| He et al. 2021 (33) | Yes | Yes | Yes | Yes | Yes | Yes | Yes | NA | NA |
| Huang et al. 2020 (34) | Yes | Yes | N/A | Yes | Yes | Yes | Yes | NA | NA |
| Huang et al. 2021 (35) | Yes | Yes | N/A | Yes | Yes | Yes | Yes | NA | NA |
| Hughes et al. 2020 (36) | Yes | Yes | Yes | Yes | Yes | Yes | Yes | NA | NA |
| Hunieres et al. 2021. (37) | Yes | Yes | Yes | Yes | Yes | Yes | Yes | NA | NA |
| Karaba et al. 2021(38) | Yes | Yes | Yes | Yes | Yes | Yes | Yes | NA | NA |
| Karami et al. 2021(39) | Yes | Yes | Yes | Yes | Yes | Yes | Yes | NA | NA |
| Kimming et al. 2020(40) | Yes | Yes | N/A | Yes | Yes | Yes | Yes | NA | NA |
| Kolenda et al. (41) | Yes | Yes | Yes | Yes | Yes | Yes | Yes | NA | NA |
| Lardaro et al. 2021 (42) | Yes | Yes | N/A | Yes | Yes | Yes | Yes | NA | NA |
| Li et al. 2020 (43) | Yes | Yes | N/A | Yes | Yes | Yes | Yes | NA | NA |
| Li et al. 2020 (a) (44) | Yes | Yes | N/A | Yes | Yes | Yes | Yes | NA | NA |
| Liu et al. 2020 (45) | Yes | Yes | N/A | Yes | Yes | Yes | Yes | NA | NA |
| Liu et al. 2021 (46) | Yes | Yes | N/A | Yes | Yes | Yes | Yes | NA | NA |
| Liu et al. 2021 (47) | Yes | Yes | N/A | Yes | Yes | Yes | Yes | NA | NA |
| Mady et a. 2020 (48) | Yes | Yes | N/A | Yes | Yes | Yes | Yes | NA | NA |
| Mahmoudi et al. 2020 (49) | Yes | Yes | N/A | Yes | Yes | Yes | Yes | NA | NA |
| Mason et al 2021. (50) | Yes | Yes | Yes | Yes | Yes | Yes | Yes | NA | NA |
| Mousav Movahed et al. 2021 (51) | Yes | Yes | N/A | Yes | Yes | Yes | Yes | NA | NA |
| Nassir et al. 2021(52) | Yes | Yes | N/A | Yes | Yes | Yes | Yes | NA | NA |
| Nebreda et al. 2020 (53) | Yes | Yes | N/A | Yes | Yes | Yes | Yes | NA | NA |
| Pulia et al. 2021 (54) | Yes | Yes | Yes | Yes | Yes | Yes | Yes | NA | NA |
| Quatuccio et al. 2020(55) | Yes | Yes | N/A | Yes | Yes | Yes | Yes | NA | NA |
| Richardson et al. 2020(56) | Yes | Yes | Yes | Yes | Yes | Yes | Yes | Yes | Yes |
| Rippa et al. 2021 (57) | Yes | Yes | N/A | Yes | Yes | Yes | Yes | NA | NA |
| Rothe et al. 2020 (58) | Yes | Yes | N/A | Yes | Yes | Yes | Yes | NA | NA |
| Seaton et al. 2020 (59) | Yes | Yes | Yes | Yes | Yes | Yes | Yes | NA | NA |
| Shah et al. 2020 (60) | Yes | Yes | N/A | Yes | Yes | Yes | Yes | NA | NA |
| Shao et al. 2020 (61) | Yes | Yes | Yes | Yes | Yes | Yes | Yes | NA | NA |
| [Sharifipour](https://bmcinfectdis.biomedcentral.com/articles/10.1186/s12879-020-05374-z#auth-Ehsan-Sharifipour) et al. 2020 (62) | Yes | Yes | N/A | Yes | Yes | Yes | Yes | NA | NA |
| Silva et al. 2021(63) | Yes | Yes | N/A | Yes | Yes | Yes | Yes | NA | NA |
| Singh et al. 2021 (64) | Yes | Yes | N/A | Yes | Yes | Yes | Yes | NA | NA |
| Soogard et al. 2021 (65) | Yes | Yes | N/A | Yes | Yes | Yes | Yes | NA | NA |
| Staub et al. 2021 (66) | Yes | Yes | N/A | Yes | Yes | Yes | Yes | NA | NA |
| Stevens et al. 2021 (67) | Yes | Yes | N/A | Yes | Yes | Yes | Yes | NA | NA |
| Tang et al. 2021 (68) | Yes | Yes | N/A | Yes | Yes | Yes | Yes | NA | NA |
| Thelen et al. 2021 (69) | Yes | Yes | Yes | Yes | Yes | Yes | Yes | NA | NA |
| Towsend et al. 2020 (70) | Yes | Yes | Yes | Yes | Yes | Yes | Yes | NA | NA |
| Vanhomwegen et al. 2021(71) | Yes | Yes | N/A | Yes | Yes | Yes | Yes | NA | NA |
| Vaughn et al. 2021 (72) | Yes | Yes | N/A | Yes | Yes | Yes | Yes | NA | NA |
| Wan et al. 2020 (73) | Yes | Yes | N/A | Yes | Yes | Yes | Yes | NA | NA |
| Wang et al. 2020 (74) | Yes | Yes | N/A | Yes | Yes | Yes | Yes | NA | NA |
| Wang et al. 2020 (75) | Yes | Yes | N/A | Yes | Yes | Yes | Yes | NA | NA |
| Wang et al. 2021(76) | Yes | Yes | Yes | Yes | Yes | Yes | Yes | NA | NA |
| Xu et al. 2021(77) | Yes | Yes | N/A | Yes | Yes | Yes | Yes | NA | NA |
| Yang et al. 2020 (78) | Yes | Yes | N/A | Yes | Yes | Yes | Yes | NA | NA |
| Zhang et al. 2020 (79) | Yes | Yes | N/A | Yes | Yes | Yes | Yes | NA | NA |
| Zhang et al. 2020 (80) | Yes | Yes | N/A | Yes | Yes | Yes | Yes | NA | NA |
| Zhang et al 2020 (81) | Yes | Yes | N/A | Yes | Yes | Yes | Yes | NA | NA |
| Zhang et al. (82) | Yes | Yes | N/A | Yes | Yes | Yes | Yes | NA | NA |
| Zhang et al 2020 (83) | Yes | Yes | N/A | Yes | Yes | Yes | Yes | NA | NA |
| Zhang et al 2020 (84) | Yes | Yes | N/A | Yes | Yes | Yes | Yes | NA | NA |
| Zhao et al 2020 (85) | Yes | Yes | N/A | Yes | Yes | Yes | Yes | NA | NA |

* NA, not applicable

**MATERIAL S4: INCLUDED STUDIES REFERENCES**

1. Alharthy A, Aletreby W, Faqihi F, Balhamar A, Alaklobi F, Alanezi K, et al. Clinical Characteristics and Predictors of 28-Day Mortality in 352 Critically Ill Patients with COVID-19: A Retrospective Study. J Epidemiol Glob Health. 2021;11(1):98-104.

2. Allou N, Larsen K, Dubernet A, Traversier N, Masse L, Foch E, et al. Co-infection in patients with hypoxemic pneumonia due to COVID-19 in Reunion Island. Medicine (Baltimore). 2021;100(4):e24524.

3. Amit M, Sorkin A, Chen J, Cohen B, Karol D, Tsur AM, et al. Clinical Course and Outcomes of Severe Covid-19: A National Scale Study. Journal of clinical medicine. 2020;9(7):2282.

4. Asmarawati TP, Rosyid AN, Suryantoro SD, Mahdi BA, Windradi C, Wulaningrum PA, et al. The clinical impact of bacterial co-infection among moderate, severe and critically ill COVID-19 patients in the second referral hospital in Surabaya. F1000Res. 2021;10:113.

5. Aydin Bahat K, Parmaksiz E, Sert S. The clinical characteristics and course of COVID-19 in hemodialysis patients. Hemodialysis International. 2020;24(4):534-40.

6. Balena F, Bavaro DF, Fabrizio C, Bottalico IF, Calamo A, Santoro CR, et al. Tocilizumab and corticosteroids for COVID-19 treatment in elderly patients. JOURNAL OF GERONTOLOGY AND GERIATRICS. 2020;68(04 Special):197-203.

7. Baraboutis IG, Gargalianos P, Aggelonidou E, Adraktas A, Collaborators. Initial Real-Life Experience from a Designated COVID-19 Centre in Athens, Greece: a Proposed Therapeutic Algorithm. SN comprehensive clinical medicine. 2020:1-5.

8. Bardi T, Pintado V, Gomez-Rojo M, Escudero-Sanchez R, Azzam Lopez A, Diez-Remesal Y, et al. Nosocomial infections associated to COVID-19 in the intensive care unit: clinical characteristics and outcome. Eur J Clin Microbiol Infect Dis. 2021;40(3):495-502.

9. Barrasa H, Rello J, Tejada S, Martin A, Balziskueta G, Vinuesa C, et al. SARS-Cov-2 in Spanish Intensive Care: Early Experience with 15-day Survival In Vitoria. Anaesthesia, critical care & pain medicine. 2020;9.

10. Mazin B, AbdulEllah A, Ali A, Layan A, Abdulaziz A, Fahad A, et al. Clinical Characteristics and Outcome of Hospitalized COVID-19 Patients in a MERS-CoV Endemic Area. Journal of Epidemiology and Global Health. 2020;10(3):214-21.

11. Baskaran V, Lawrence H, Lansbury LE, Webb K, Safavi S, Zainuddin NI, et al. Co-infection in critically ill patients with COVID-19: an observational cohort study from England. J Med Microbiol. 2021;70(4).

12. Bhatt PJ, Shiau S, Brunetti L, Xie Y, Solanki K, Khalid S, et al. Risk Factors and Outcomes of Hospitalized Patients With Severe Coronavirus Disease 2019 (COVID-19) and Secondary Bloodstream Infections: A Multicenter Case-Control Study. Clin Infect Dis. 2021;72(12):e995-e1003.

13. Buckner FS, McCulloch DJ, Atluri V, Blain M, McGuffin SA, Nalla AK, et al. Clinical Features and Outcomes of 105 Hospitalized Patients With COVID-19 in Seattle, Washington. Clin Infect Dis. 2020;71(16):2167-73.

14. Chen T, Dai Z, Mo P, Li X, Ma Z, Song S, et al. Clinical characteristics and outcomes of older patients with coronavirus disease 2019 (COVID-19) in Wuhan, China (2019): a single-centered, retrospective study. The journals of gerontology Series A, Biological sciences and medical sciences. 2020;11.

15. Chen S, Zhu Q, Xiao Y, Wu C, Jiang Z, Liu L, et al. Clinical and etiological analysis of co-infections and secondary infections in COVID-19 patients: An observational study. Clin Respir J. 2021;15(7):815-25.

16. Cheng LS, Chau SK, Tso EY, Tsang SW, Li IY, Wong BK, et al. Bacterial co-infections and antibiotic prescribing practice in adults with COVID-19: experience from a single hospital cluster. Ther Adv Infect Dis. 2020;7:2049936120978095.

17. Chengyi HU, Lushan X, Hongbo Z, Yanpei Z, Wenfeng Z, Li L, et al. Effect of hypertension on outcomes of patients with COVID-19. Nan fang yi ke da xue xue bao = Journal of Southern Medical University. 2020;40(11):1537-42.

18. Chong WH, Chieng H, Tiwari A, Beegle S, Feustel PJ, Ghalib S, et al. Incidence and Risk Factors for Secondary Pulmonary Infections in Patients Hospitalized With Coronavirus Disease 2019 Pneumonia. Am J Med Sci. 2021.

19. Choubey A, Cawley P, Miller K, Sagar D. Retrospective review analysis of COVID-19 patients co-infected with Mycoplasma pneumoniae. Lung India. 2021;38(7):S22-S6.

20. Contou D, Claudinon A, Pajot O, Micaëlo M, Longuet Flandre P, Dubert M, et al. Bacterial and viral co-infections in patients with severe SARS-CoV-2 pneumonia admitted to a French ICU. Annals of Intensive Care. 2020;10(1):119.

21. D'Onofrio V, Van Steenkiste E, Meersman A, Waumans L, Cartuyvels R, Van Halem K, et al. Differentiating influenza from COVID-19 in patients presenting with suspected sepsis. Eur J Clin Microbiol Infect Dis. 2021;40(5):987-95.

22. Desai A, Santonocito OG, Caltagirone G, Kogan M, Ghetti F, Donadoni I, et al. Effectiveness of Streptococcus Pneumoniae Urinary Antigen Testing in Decreasing Mortality of COVID-19 Co-Infected Patients: A Clinical Investigation. Medicina (Kaunas). 2020;56(11).

23. Dolci A, Robbiano C, Aloisio E, Chibireva M, Serafini L, Falvella FS, et al. Searching for a role of procalcitonin determination in COVID-19: a study on a selected cohort of hospitalized patients. Clin Chem Lab Med. 2020;59(2):433-40.

24. Ekadashi R, Garg VK, Daisy B, Richa S, Rajat V, Vedprakash M, et al. Prevalence of bloodstream infections and their etiology in COVID-19 patients admitted in a tertiary care hospital in Jaipur. Indian Journal of Critical Care Medicine. 2021;25(4):369-73.

25. Elabbadi A, Turpin M, Gerotziafas GT, Teulier M, Voiriot G, Fartoukh M. Bacterial coinfection in critically ill COVID-19 patients with severe pneumonia. Infection. 2021;49(3):559-62.

26. Falcone M, Tiseo G, Giordano C, Leonildi A, Menichini M, Vecchione A, et al. Predictors of hospital-acquired bacterial and fungal superinfections in COVID-19: a prospective observational study. J Antimicrob Chemother. 2021;76(4):1078-84.

27. Fan L, Liu H, Li N, Liu C, Gu Y, Liu Y, et al. Medical treatment of 55 patients with COVID-19 from seven cities in northeast China who fully recovered: A single-center, retrospective, observational study. Medicine (Baltimore). 2021;100(2):e23923.

28. Garcia-Vidal C, Sanjuan G, Moreno-García E, Puerta-Alcalde P, Garcia-Pouton N, Chumbita M, et al. Incidence of co-infections and superinfections in hospitalized patients with COVID-19: a retrospective cohort study. Clin Microbiol Infect. 2021;27(1):83-8.

29. Gayam V, Konala VM, Naramala S, Garlapati PR, Merghani MA, Regmi N, et al. Presenting characteristics, comorbidities, and outcomes of patients coinfected with COVID-19 and Mycoplasma pneumoniae in the USA. Journal of medical virology. 2020;92(10):2181-7.

30. Goncalves Mendes Neto A, Lo KB, Wattoo A, Salacup G, Pelayo J, DeJoy R, 3rd, et al. Bacterial infections and patterns of antibiotic use in patients with COVID-19. J Med Virol. 2021;93(3):1489-95.

31. Guan J, Wei X, Qin S, Liu X, Jiang Y, Chen Y, et al. Continuous tracking of COVID-19 patients' immune status. International immunopharmacology. 2020;89(Pt A):107034-.

32. He F, Xia X, Nie D, Yang H, Jiang Y, Huo X, et al. Respiratory bacterial pathogen spectrum among COVID-19 infected and non-COVID-19 virus infected pneumonia patients. Diagnostic microbiology and infectious disease. 2020;98(4):115199-.

33. He S, Liu W, Jiang M, Huang P, Xiang Z, Deng D, et al. Clinical characteristics of COVID-19 patients with clinically diagnosed bacterial co-infection: A multi-center study. PloS one. 2021;16(4):e0249668-e.

34. Huang C, Wang Y, Li X, Ren L, Zhao J, Hu Y, et al. Clinical features of patients infected with 2019 novel coronavirus in Wuhan, China. Lancet. 2020;395(10223):497-506.

35. Huang E, Isonaka S, Yang H, Salce E, Rosales E, Jordan SC. Tocilizumab treatment in critically ill patients with COVID-19: A retrospective observational study. International journal of infectious diseases : IJID : official publication of the International Society for Infectious Diseases. 2021;105:245-51.

36. Hughes S, Troise O, Donaldson H, Mughal N, Moore LSP. Bacterial and fungal coinfection among hospitalized patients with COVID-19: a retrospective cohort study in a UK secondary-care setting. Clin Microbiol Infect. 2020;26(10):1395-9.

37. d'Humières C, Patrier J, Lortat-Jacob B, Tran-Dinh A, Chemali L, Maataoui N, et al. Two original observations concerning bacterial infections in COVID-19 patients hospitalized in intensive care units during the first wave of the epidemic in France. PLoS One. 2021;16(4):e0250728.

38. Karaba SM, Jones G, Helsel T, Smith LL, Avery R, Dzintars K, et al. Prevalence of Co-infection at the Time of Hospital Admission in COVID-19 Patients, A Multicenter Study. Open Forum Infectious Diseases. 2021;8(1):ofaa578.

39. Karami Z, Knoop BT, Dofferhoff ASM, Blaauw MJT, Janssen NA, van Apeldoorn M, et al. Few bacterial co-infections but frequent empiric antibiotic use in the early phase of hospitalized patients with COVID-19: results from a multicentre retrospective cohort study in The Netherlands. Infect Dis (Lond). 2021;53(2):102-10.

40. Kimmig LM, Wu D, Gold M, Pettit NN, Pitrak D, Mueller J, et al. IL-6 Inhibition in Critically Ill COVID-19 Patients Is Associated With Increased Secondary Infections. Frontiers in medicine. 2020;7:583897-.

41. Kolenda C, Ranc AG, Boisset S, Caspar Y, Carricajo A, Souche A, et al. Assessment of Respiratory Bacterial Coinfections Among Severe Acute Respiratory Syndrome Coronavirus 2-Positive Patients Hospitalized in Intensive Care Units Using Conventional Culture and BioFire, FilmArray Pneumonia Panel Plus Assay. Open Forum Infect Dis. 2020;7(11):ofaa484.

42. Lardaro T, Wang AZ, Bucca A, Croft A, Glober N, Holt DB, et al. Characteristics of COVID-19 patients with bacterial coinfection admitted to the hospital from the emergency department in a large regional healthcare system. Journal of medical virology. 2021;93(5):2883-9.

43. Li R, Tian J, Yang F, Lv L, Yu J, Sun G, et al. Clinical characteristics of 225 patients with COVID-19 in a tertiary Hospital near Wuhan, China. Journal of Clinical Virology. 2020;127(104363).

44. Li J, Wang J, Yang Y, Cai P, Cao J, Cai X, et al. Etiology and antimicrobial resistance of secondary bacterial infections in patients hospitalized with COVID-19 in Wuhan, China: a retrospective analysis. Antimicrobial Resistance & Infection Control. 2020;9(1):153.

45. Liu F, Li L, Xu M, Wu J, Luo D, Zhu Y, et al. Prognostic value of interleukin-6, C-reactive protein, and procalcitonin in patients with COVID-19. Journal of Clinical Virology. 2020;127(104370).

46. Liu L, Lei X, Xiao X, Yang J, Li J, Ji M, et al. Epidemiological and Clinical Characteristics of Patients With Coronavirus Disease-2019 in Shiyan City, China. Frontiers in cellular and infection microbiology. 2020;10:284-.

47. Liu C, Wen Y, Wan W, Lei J, Jiang X. Clinical characteristics and antibiotics treatment in suspected bacterial infection patients with COVID-19. International immunopharmacology. 2021;90:107157-.

48. Mady A, Aletreby W, Abdulrahman B, Lhmdi M, Noor AM, Alqahtani SA, et al. Tocilizumab in the treatment of rapidly evolving COVID-19 pneumonia and multifaceted critical illness: A retrospective case series. Ann Med Surg (Lond). 2020;60:417-24.

49. Mahmoudi H. Bacterial co-infections and antibiotic resistance in patients with COVID-19. GMS hygiene and infection control. 2020;15:Doc35-Doc.

50. Mason CY, Kanitkar T, Richardson CJ, Lanzman M, Stone Z, Mahungu T, et al. Exclusion of bacterial co-infection in COVID-19 using baseline inflammatory markers and their response to antibiotics. Journal of Antimicrobial Chemotherapy. 2021;76(5):1323-31.

51. Mousavi Movahed SM, Akhavizadegan H, Dolatkhani F, Nejadghaderi SA, Aghajani F, Faghir Gangi M, et al. Different incidences of acute kidney injury (AKI) and outcomes in COVID-19 patients with and without non-azithromycin antibiotics: A retrospective study. J Med Virol. 2021;93(7):4411-9.

52. Nasir N, Rehman F, Omair SF. Risk factors for bacterial infections in patients with moderate to severe COVID-19: A case-control study. Journal of medical virology. 2021;93(7):4564-9.

53. Nebreda-Mayoral T, Miguel-Gómez MA, March-Rosselló GA, Puente-Fuertes L, Cantón-Benito E, Martínez-García AM, et al. Bacterial/fungal infection in hospitalized patients with COVID-19 in a tertiary hospital in the Community of Castilla y León, Spain. Enferm Infecc Microbiol Clin (Engl Ed). 2020.

54. Pulia MS, Wolf I, Schwei RJ, Chen D, Lepak AJ, Schulz LT, et al. Antibiotic prescribing patterns for coronavirus disease 2019 (COVID-19) in two emergency departments with rapid procalcitonin. Infection control and hospital epidemiology. 2021;42(3):359-61.

55. Quartuccio L, Sonaglia A, McGonagle D, Fabris M, Peghin M, Pecori D, et al. Profiling COVID-19 pneumonia progressing into the cytokine storm syndrome: Results from a single Italian Centre study on tocilizumab versus standard of care. J Clin Virol. 2020;129:104444-.

56. Richardson S, Hirsch JS, Narasimhan M, Crawford JM, McGinn T, Davidson KW, et al. Presenting Characteristics, Comorbidities, and Outcomes Among 5700 Patients Hospitalized With COVID-19 in the New York City Area. Jama. 2020;22.

57. Ripa M, Galli L, Poli A, Oltolini C, Spagnuolo V, Mastrangelo A, et al. Secondary infections in patients hospitalized with COVID-19: incidence and predictive factors. Clin Microbiol Infect. 2021;27(3):451-7.

58. Rothe K, Feihl S, Schneider J, Wallnöfer F, Wurst M, Lukas M, et al. Rates of bacterial co-infections and antimicrobial use in COVID-19 patients: a retrospective cohort study in light of antibiotic stewardship. European journal of clinical microbiology & infectious diseases : official publication of the European Society of Clinical Microbiology. 2021;40(4):859-69.

59. Seaton RA, Gibbons CL, Cooper L, Malcolm W, McKinney R, Dundas S, et al. Survey of antibiotic and antifungal prescribing in patients with suspected and confirmed COVID-19 in Scottish hospitals. The Journal of infection. 2020;81(6):952-60.

60. Shah SJ, Barish PN, Prasad PA, Kistler A, Neff N, Kamm J, et al. Clinical features, diagnostics, and outcomes of patients presenting with acute respiratory illness: A retrospective cohort study of patients with and without COVID-19. eClinicalMedicine. 2020;27.

61. Shao S, Zhao Z, Wang F, Chang D, Liu Y, Liu S, et al. Risk factors associated with disease aggravation among 126 hospitalized patients with COVID-19 in different places in China: A retrospective observational study. Medicine. 2020;99(45):e22971-e.

62. Sharifipour E, Shams S, Esmkhani M, Khodadadi J, Fotouhi-Ardakani R, Koohpaei A, et al. Evaluation of bacterial co-infections of the respiratory tract in COVID-19 patients admitted to ICU. BMC Infectious Diseases. 2020;20(1):646.

63. Silva DL, Lima CM, Magalhães VCR, Baltazar LM, Peres NTA, Caligiorne RB, et al. Fungal and bacterial coinfections increase mortality of severely ill COVID-19 patients. Journal of Hospital Infection. 2021;113:145-54.

64. Singh V, Upadhyay P, Reddy J, Granger J. SARS-CoV-2 respiratory co-infections: Incidence of viral and bacterial co-pathogens. International journal of infectious diseases : IJID : official publication of the International Society for Infectious Diseases. 2021;105:617-20.

65. Søgaard KK, Baettig V, Osthoff M, Marsch S, Leuzinger K, Schweitzer M, et al. Community-acquired and hospital-acquired respiratory tract infection and bloodstream infection in patients hospitalized with COVID-19 pneumonia. Journal of Intensive Care. 2021;9(1):10.

66. Staub MB, Beaulieu RM, Graves J, Nelson GE. Changes in antimicrobial utilization during the coronavirus disease 2019 (COVID-19) pandemic after implementation of a multispecialty clinical guidance team. Infection control and hospital epidemiology. 2021;42(7):810-6.

67. Stevens RW, Jensen K, O'Horo JC, Shah A. Antimicrobial prescribing practices at a tertiary-care center in patients diagnosed with COVID-19 across the continuum of care. Infection control and hospital epidemiology. 2021;42(1):89-92.

68. Tang M-L, Li Y-Q, Chen X, Lin H, Jiang Z-C, Gu D-L, et al. Co-Infection with Common Respiratory Pathogens and SARS-CoV-2 in Patients with COVID-19 Pneumonia and Laboratory Biochemistry Findings: A Retrospective Cross-Sectional Study of 78 Patients from a Single Center in China. Med Sci Monit. 2021;27:e929783-e.

69. Thelen JM, Buenen AG, van Apeldoorn M, Wertheim HF, Hermans MHA, Wever PC. Community-acquired bacteraemia in COVID-19 in comparison to influenza A and influenza B: a retrospective cohort study. BMC Infectious Diseases. 2021;21(1):199.

70. Townsend L, Hughes G, Kerr C, Kelly M, O'Connor R, Sweeney E, et al. Bacterial pneumonia coinfection and antimicrobial therapy duration in SARS-CoV-2 (COVID-19) infection. JAC Antimicrob Resist. 2020;2(3):dlaa071.

71. Vanhomwegen C, Veliziotis I, Malinverni S, Konopnicki D, Dechamps P, Claus M, et al. Procalcitonin accurately predicts mortality but not bacterial infection in COVID-19 patients admitted to intensive care unit. Irish journal of medical science. 2021;190(4):1649-52.

72. Vaughn VM, Gandhi TN, Petty LA, Patel PK, Prescott HC, Malani AN, et al. Empiric Antibacterial Therapy and Community-onset Bacterial Coinfection in Patients Hospitalized With Coronavirus Disease 2019 (COVID-19): A Multi-hospital Cohort Study. Clinical Infectious Diseases. 2021;72(10):e533-e41.

73. Wan S, Xiang Y, Fang W, Zheng Y, Li B, Hu Y, et al. Clinical features and treatment of COVID-19 patients in northeast Chongqing. Journal of Medical Virology. 2020.

74. Wang F, Yang Y, Dong K, Yan Y, Zhang S, Ren H, et al. Clinical characteristics of 28 patients with diabetes and COVID-19 in Wuhan, China. Endocr Pract. 2020.

75. Wang L, He W, Yu X, Hu D, Bao M, Liu H, et al. Coronavirus disease 2019 in elderly patients: Characteristics and prognostic factors based on 4-week follow-up. Journal of Infection. 2020;80(6):639-45.

76. Wang L, Amin AK, Khanna P, Aali A, McGregor A, Bassett P, et al. An observational cohort study of bacterial co-infection and implications for empirical antibiotic therapy in patients presenting with COVID-19 to hospitals in North West London. Journal of Antimicrobial Chemotherapy (JAC). 2021;76(3):796-803.

77. Xu J, Zhang J, Li Y, Pan Y, Liu T, Zhao J, et al. Clinical features for severely and critically ill patients with COVID-19 in shandong: A retrospective cohort study. Therapeutics and Clinical Risk Management. 2021;17:9-21.

78. Yang G, Tan Z, Zhou L, Yang M, Peng L, Liu J, et al. Effects Of ARBs And ACEIs On Virus Infection, Inflammatory Status And Clinical Outcomes In COVID-19 Patients With Hypertension: A Single Center Retrospective Study. Hypertension. 2020.

79. Zhang G, Hu C, Luo L, Fang F, Chen Y, Li J, et al. Clinical features and short-term outcomes of 221 patients with COVID-19 in Wuhan, China. Journal of Clinical Virology. 2020;127(104364).

80. Zhang JJ, Dong X, Cao YY, Yuan YD, Yang YB, Yan YQ, et al. Clinical characteristics of 140 patients infected with SARS-CoV-2 in Wuhan, China. Allergy: European Journal of Allergy and Clinical Immunology. 2020.

81. Zhang H, Zhang Y, Wu J, Li Y, Zhou X, Chen S, et al. Risks and features of secondary infections in severe and critical ill COVID-19 patients. Emerging Microbes and Infections. 2020;9(1):1958-64.

82. Zhang W, Hou W, Jin R, Liang L, Xu B, Hu Z. Clinical characteristics and outcomes in elderly with coronavirus disease 2019 in Beijing, China: a retrospective cohort study. Intern Emerg Med. 2021;16(4):875-82.

83. Zhang L, Fan H, Zhang H, Tao X, Cheng S, Chen J, et al. Retrospective analysis of clinical features in 134 coronavirus disease 2019 cases. Epidemiology and Infection. 2020.

84. Zhang Y, Xiao LS, Li P, Zhu H, Hu C, Zhang WF, et al. Clinical Characteristics of Patients With Progressive and Non-progressive Coronavirus Disease 2019: Evidence From 365 Hospitalised Patients in Honghu and Nanchang, China. Front Med (Lausanne). 2020;7:556818.

85. Zhao M, Wang M, Zhang J, Gu J, Zhang P, Xu Y, et al. Comparison of clinical characteristics and outcomes of patients with coronavirus disease 2019 at different ages. Aging (Albany NY). 2020;12(11):10070-86.
